# Aptamer-targeted anti-miR RNA construct based on 3WJ as a new approach for the treatment of chronic kidney disease in an experimental model

**DOI:** 10.1038/s41434-025-00544-7

**Published:** 2025-06-14

**Authors:** Aisha H. A. Alsenousy, Sara A. Sharker, Mennatallah A. Gowayed, Samar S. Elblehi, Maher A. Kamel

**Affiliations:** 1 Department of Biochemistry, Medical Research Institute, University of Alexandria, Alexandria, Egypt; 2 Department of Pharmacology and Therapeutics, Faculty of Pharmacy and Drug Manufacturing, Pharos University in Alexandria, Alexandria, Egypt; 3 Department of Pathology, Faculty of Veterinary Medicine, Alexandria University, Alexandria, Egypt; 4 Research Projects unit, Pharos University in Alexandria, 21648 Alexandria, Egypt

**Keywords:** Antagomir and RNA sponge, Nanoparticles

## Abstract

The treatment of chronic disease (CKD) is a great challenge in healthcare that requires an innovative approach to address its complex nature. RNA nanotechnology has emerged rapidly and received attention in the last few years because of its significant aptitude for therapies. Hence, the present study aimed to design, construct, and characterize a multifunctional (anti-miR-34a DNA aptamer-kidney targeted) RNA nanoparticle (RNPs) based on bacteriophage phi29 packaging RNA three-way junction (pRNA-3WJ), and then explore their in vivo toxicity and therapeutic potentials in mice model of CKD. After confirming the safety and specific targeting capability of the prepared core 3WJ (3WJ) and the therapeutic 3WJ (3WJ-Kapt/anti-miR-34a) RNPs to renal tissue using healthy mice, CKD was induced in C57BL/6 mice using adenine. CKD mice were treated with a single intravenous injection of 3WJ or 3WJ-Kapt/anti-miR-34a. Every week, 5 mice of each group were selected randomly for sample collection for 4 weeks post-treatment. The anti-miR-34a 3WJ-RNPs have shown stability, safety, and efficacy in renal targeting using DNA aptamer, by targeting miR-34a in renal tissue, 3WJ-Kapt/anti-miR-34a suppressed profibrotic gene expression and induced anti-fibrotic pathways’ expression. Our present study provides preliminary and pioneering evidence for the promising treatment of renal fibrosis and CKD through targeting miR-34a in the renal tissue by 3WJ-RNPs.

**Figure:**
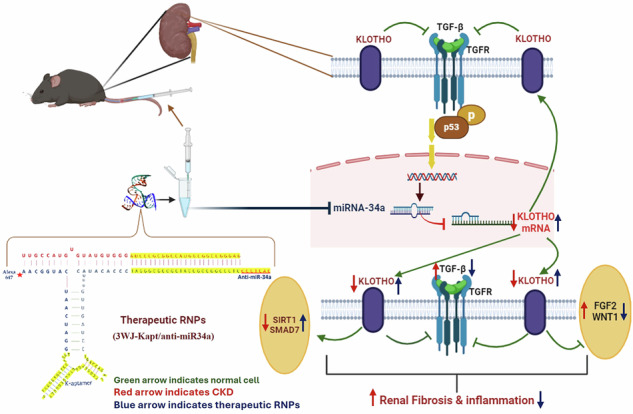
The CKD mice showed marked time-dependent up-regulation of the renal profibrotic pathways, including TGF-β, FGF2, and WNT/β-catenin pathways. The same mice showed suppressed renal expression of the antifibrotic pathways, including α and β Klotho, SMAD7, and SIRT1. The prepared anti-miR-34a 3WJ-RNPs have shown stability, safety, and efficacy in renal targeting using DNA aptamer. By targeting miR-34a in renal tissue, 3WJ-Kapt/anti-miR-34a suppressed profibrotic gene expression and induced anti-fibrotic pathways’ expression. Our present study provides preliminary and pioneer evidence for the promising treatment of renal fibrosis and CKD through targeting miR-34a in the renal tissue by 3WJ-RNPs.

The CKD mice showed marked time-dependent up-regulation of the renal profibrotic pathways, including TGF-β, FGF2, and WNT/β-catenin pathways. The same mice showed suppressed renal expression of the antifibrotic pathways, including α and β Klotho, SMAD7, and SIRT1. The prepared anti-miR-34a 3WJ-RNPs have shown stability, safety, and efficacy in renal targeting using DNA aptamer. By targeting miR-34a in renal tissue, 3WJ-Kapt/anti-miR-34a suppressed profibrotic gene expression and induced anti-fibrotic pathways’ expression. Our present study provides preliminary and pioneer evidence for the promising treatment of renal fibrosis and CKD through targeting miR-34a in the renal tissue by 3WJ-RNPs.

## Introduction

Chronic kidney disease (CKD) is a slowly progressive systemic disease that results in an irreversible long-term loss of kidney function [1, 2]. CKD is a life-threatening disease that is becoming more common, involving about 10% of the global population. According to recent estimates, 800 million of the global population suffer from CKD [3, 4]. Since 1990, this prevalence has risen by around 30% [5]. The three main pathological characteristics of CKD are interstitial inflammation, tubular atrophy, and fibrosis. Functional impairment of the kidney is more strongly connected to tubulointerstitial damage than to glomerular injury, regardless of the underlying etiology [6], which sets off an inflammatory cascade, including T-cell recruitment and macrophage activation. This in turn, produces profibrotic mediators, like transforming growth factor-β (TGF-β), which is a crucial cytokine that has been of increasing concern in recent years [7], as a dysregulated TGF-β signal plays an essential role in contributing to fibrosis via promoting the extracellular matrix deposition [8].

The treatment of CKD is a great challenge in healthcare that requires an innovative approach to address its complex nature. RNA nanotechnology has emerged in recent years and continues to develop because of its potential therapeutic applications [9]. The RNA nanotechnology platform is unique compared to many other well-developed nano-delivery technologies, including liposomes, polymers, dendrimers, inorganic, and viral. Adding ligands to polyvalent RNA nanoparticles (RNPs) allows them to be delivered to target cells [10]. Because of their uniform nanoscale size, polyvalent nature, exact stoichiometry, low toxicity, low immunogenicity, and target specificity, 3WJ-RNPs have the potential to be used in clinical applications as a targeted therapeutic delivery system to treat a variety of disorders [11]. RNA instability is no longer an obstacle that can be managed through various chemical modifications. notably, RNA can be used as a medication, including anti-miRNA, siRNA, aptamer, miRNA, and ribozyme [12, 13].

Guo presented the first proof-of-concept study demonstrating the viability of RNA nanotechnology in 1998 [14]. The thermodynamic stability of the bacteriophage phi29 packaging RNA three-way junction (pRNA-3WJ) may be exploited to create multifunctional nanoparticles with therapeutic effects [15]. The phi29 pRNA-3WJ motif offers the perfect framework for creating multifunctional RNPs. The RNPs with regulated topologies, defined sizes, accurate stoichiometries, and polyvalent functionalities can be created using a bottom-up self-assembly method by integrating various functional modules into the branching region of a three-way junction (3WJ) [16]. The three branches of the 3WJ can be expanded to carry flexible functional modules that can be utilized as targeting, imaging, and therapeutic modules without compromising the system’s overall stability and structure. This allows the 3WJ to operate as a platform for target recognition systems. Because of its huge payloads and remarkable enzymatic and thermal stability, pRNA-3WJ is a valuable tool for RNA nanotechnology and the construction of nanomaterials [9].

One essential protein that shields the kidneys is Klotho. The deficiency of Klotho is linked to the pathogenesis, development, and progression of CKD as well as extrarenal complications. Its deficiency enhances renal tubular and vascular cell senescence induced by oxidative stress. It also suppresses TGF-β activity, TGF-receptor II, and Wingless-related integration site (WNT) signaling activity promoting renal fibrosis [17, 18]. The kidneys produce sirtuin 1 (SIRT1), which mediates several physiological processes and protects and maintains appropriate kidney cell function. SIRT1 suppresses TNF-dependent transactivation of Nuclear factor kappa B (NF-kB), suppressing the expression of many pro-inflammatory genes and Tumor necrosis factor alpha (TNF-α) -induced cytokine production in fibroblast cells. SIRT1 also interacts with TGF-β signaling to provide an anti-fibrosis impact in CKD [19].

Renal fibrosis is the final common pathway of all progressive renal diseases, so it is an interesting target for CKD treatment. The TGF-β as a “core signaling pathway” was found to regulate several microRNAs (miRs), which function as downstream mediators of distinct pro-fibrotic effects. While overexpression of miR-34 induces renal fibrosis, its downregulation has been shown to decrease renal fibrosis [7]. For this reason, the present study aimed to design, prepare, and characterize multifunctioning (antimir-34a and DNA aptamer-kidney targeted) RNA nanoparticle (RNPs) based on bacteriophage phi29 packaging RNA three-way junction (pRNA-3WJ), and then explore their in vivo toxicity and therapeutic potentials in mice model of CKD. Besides the molecular mechanisms involved in their therapeutic effect, the stability, safety, and efficient renal targeting of the prepared anti-miR-34a 3WJ-RNPs were evaluated.

## Materials and methods

### In silico phase

#### Designing the core and therapeutic three-way junction RNA nanoparticles (3WJ-RNPs)

The core 3WJ-RNPs (3WJ) were designed using the basic three single-stranded RNA (ssRNA) sequences (3WJ-a, 3WJ-b, and 3WJ-c) [20]. The sequences of these strands are presented in Table 1. Based on the sequences of the basic three ssRNA of the core 3WJ-RNPs we designed four single strands for the construction of the therapeutic 3WJ-RNPs (3WJ-Kapt/anti-miR-34a) which is aptamer-targeted to renal tissues and functionalized with an anti-miR-34a domain as follows: Strand-1: RNA sequence of 3WJ-a plus 3´-extending arm consisting of 25 ribonucleotides, Strand-2: Start from the 5´-end with anti-miR-34a hepta-deoxyribonucleotide (CACTGCC) followed by 25 Deoxyribonucleotides complementary to the extending arm of strand 1, Strand-3: RNA sequence of 3WJ-b plus 3´- extending 43 Deoxyribonucleotides of Kapt [21], Strand-4: RNA sequence of 3WJ-c.

**Table 1:** The sequences of the core three-way junction (3WJ) and the therapeutic three-way junction (3WJ-Kapt/anti-miR-34a) RNA nanoparticle strands.

Strand Name	RNA sequence 5ʹ → 3ʹ
**Strands of 3WJ**	
**3WJ-a**	UUGCCAUGUGUAUGUGGG
**3WJ-b**	CCCACAUACUUUGUUGAUCC
**3WJ-c**	GGAUCAAUCAUGGCAA
**Strands of 3WJ-Kapt/anti-miR-34a**	
**Strand-1**	UUGCCAUGUGUAUGUGGG**AUCCCGCGGCCAUGGCGGCCGGGAG**
**Strand-2**	**CACTGCCCTCCCGGCCGCCATGGCCGCGGGAT**
**Strand-3**	CCCACAUACUUUGUUGAUCC**TGACCAGACGGGGTGGGTGGGCGGGCTGTCGGCTGGTCCTCGT**
**Strand-4**	GGAUCAAUCAUGGCAA

The normal letters refer to ribonucleotides, while the **bold** letters refer to deoxyribonucleotides, and the **underlined** letters refer to the anti-miR-34a hepta-nucleotides.

The 3WJ-c strand and strand-4 were labeled with Alexa Fluor 647 to facilitate the detection of the RNPs in vivo, all U and C ribonucleotides were replaced with 2’-Fluoro (2′-F) U and C ribonucleotides and the anti-miR-34a sequence (7- deoxyribonucleotides) used as locked nucleic acid (LNA) to increase the enzymatic and thermal stability of the RNPs.The proper folding of these strands into correct 3WJ RNPs folding was predicted using the VfoldMCPX online tool (http://rna.physics.missouri.edu/vfoldMCPX2) which predicts the 2D structures of multi-strand RNA complexes [22]. The sequences of the strands with modifications were obtained from Viviantis Technologies (Malaysia).

## In vitro phase

### Stepwise bottom-up assembly of 3WJs

Each strand was prepared in the corresponding volume of Diethylpyrocarbonate water as documented in the datasheet to prepare equimolar concentrations (20 µM). Each type of 3WJs (core or therapeutic) is stepwise prepared.

### Preparation of core 3WJ

The RNPs were constructed by mixing 3WJ-b, and 3WJ-c strands after 10 min at room temperature, then 3WJ-a strand was added (Table 1) at an equal molar concentration in TMS buffer (50 mM Tris pH 8.0, 100 mM NaCl, 10 mM MgCl_2_), followed by heating to 85 °C for 5 minutes and slowly the particle size (ps), and zeta potential (zp) were then measured based on the dynamic light scattering (DLS) method, using the Malvern Zetasizer Ultra. cooled over 40 minutes to 4 °C on a thermal cycler [20].

### Preparation of 3WJ-Kapt/anti-miR-34a

The RNPs were constructed by mixing strands (3 and 4) after 10 min at room temperature, then strands (1 and 2) were added (Table 1) at an equal molar concentration in TMS buffer, followed by heating to 85 °C for 5 minutes and slowly cooled over 40 minutes to 4 °C on a thermal cycler [20].

### Characterization of the prepared RNA nanoparticles

The prepared RNA nanoparticles were characterized by using agarose gel electrophoresis, thermal stability by thermal cycler (Tm), The 3WJs were dissolved in diethylpyrocarbonate-treated water to a final concentration of 1–2 μg/μl.

## In vivo phase

The in vivo experiments were conducted on 97 c57bl/6 mice with an average weight of 20-25 g obtained from the animal house of the Medical Research Institute, Alexandria University, Egypt. The sample size was decided using power calculations and don’t used the randomization and blinding methods. All mice had free access to food and water with a 12:12 hour light/dark cycle and constant environmental conditions before experimentation. The use of experimental animals in the study protocol was carried out by the Ethical Guidelines of The Institutional Animal Care and Use Committee (IACUC) at Alexandria University, Egypt (approval no. AU012237921).

In vivo experiments were divided into two parts: part 1, a toxicity study to examine the safety of the prepared 3WJ, and 3WJ-Kapt/anti-miR-34a RNPs on the control mice. Part 2: a therapeutic study to examine the targeting and therapeutic efficiency of the prepared 3WJ-Kapt/anti-miR-34a RNPs on the CKD mice compared with the untreated CKD mice or mice treated with 3WJ RNPs (as a vehicle).

### Explore in vivo toxicity, safety, and targeting efficiency of the prepared renal cell-targeted multifunctioning 3WJ and 3WJ-Kapt/anti-miR-34a RNPs

A toxicity study was conducted to examine the safety of the prepared 3WJ and 3WJ-Kapt/anti-miR-34a RNPs on healthy mice. 19 normal C57BL/6 mice were divided into three groups: untreated healthy control, 3WJ-treated, and 3WJ-Kapt/anti-miR-34a-treated. All mice were injected once intravenously in the tail vein with 200 µL of TMS, 3WJ (50 µg/kg), or 3WJ-Kapt/anti-miR-34a (50 µg/kg), respectively. After 24 hours two mice from 3WJ-Kapt/anti-miR-34areated groups were sacrificed to obtain different organs for the biodistribution assessment using a confocal microscope (LEICA DMI8, Germany). The remaining mice were followed up for 4 weeks then the mice were sacrificed, and blood was obtained for separation of serum and both kidneys were dissected out. The serum was used for the assessment of liver and kidney functions and the tissues were used for assessing inflammatory and oxidative markers.

### Therapeutic study

This study involved 80 C57BL/6 mice, 20 normal mice, and 60 mice with CKD. The CKD was induced by oral administration of adenine at a dose of 50 mg/kg body weight (dissolved in 0.5% Carboxymethyl cellulose, CMC) by oral gavage for 28 days [23]. To confirm the establishment of CKD, a serum sample was withdrawn on day 29 after induction for the measurement of urea and creatinine levels. A CKD was considered if the urea level reached more than 100 mg/dL and the creatinine level was higher than 1 mg/dl [24].

### Experimental design

The mice were divided into two main groups: The control group (20 mice) that received single i.v. injection in the tail vein with 200 µL saline and CKD group (60 mice). The latter was subdivided into three subgroups: Untreated CKD group: CKD mice that received no treatment, CKD + 3WJ group: CKD mice that received single i.v. injection in the tail vein with 200 µL of 3WJ (50 µg/kg) [16], and CKD + 3WJ-Kapt/anti-miR-34a group: CKD mice that received single i.v. injection in the tail vein with 200 µL of 3WJ-Kapt/anti-miR-34a (50 µg/kg) [16].

The mice had free access to food and water and were followed up for 4 weeks post-treatment. Every week 5 mice of each group were selected randomly and sacrificed under isoflurane anesthesia to obtain the blood and to dissect kidneys, liver, heart, spleen, and brain.

### Samples preparation

Blood samples were collected in 2 tubes. In a plain tube, the samples were left for 20 min at room temperature and centrifuged at 3000 ×g for 10 minutes to obtain serum for the assessment of urea, creatinine, alanine aminotransferase (ALT), aspartate aminotransferase (AST) activities phosphate, erythropoietin (EPO), Kidney Injury Molecule-1 (KIM-1), and N-acetyl-β-D-glucosaminidase (NAG). The remaining blood was collected into tubes containing EDTA as an anticoagulant to assess the hemoglobin concentration.

The left kidneys were quickly dissected out, rinsed with phosphate buffer saline (PBS), and used for fluorescence scanning and histological analysis. The right kidneys were divided into two aliquots: the first aliquot was used for total RNA extraction for subsequent gene expression analysis of miR-34a, Fibroblast Growth Factor 2 (FGF2), Suppressor of Mothers Against Decapentaplegic (SMAD7), TGF-β, β-Klotho, α-Klotho, WNT1, β-catenin, and SIRT1 (the methods and primers used were provided in the supplementary file). The second one was homogenized in RIBA buffer (1:9) and used for the determination of malondialdehyde (MDA) level, then centrifuged at 10,000×g for 10 min at 4 ^o^C, and the supernatant was stored in aliquots for subsequent determinations of total protein level by Lowry method [25], TGF-β, SMAD2, SMAD3, α-Klotho KIM-1, NAG, TNF-α, and IL-6 (the methods and sources of the used kits were provided in the supplementary file). All the other collected tissues (liver, heart, spleen, and brain) were used for fluorescence scanning to detect the biodistribution of the RNPs. Slides for histopathological settings were stained with hematoxylin and eosin (H&E). A detailed methodology for each measurement is provided in the supplementary file.

### Histopathological examination and lesion scoring

Following necropsy, tissue specimens from the kidneys were immediately fixed in phosphate-buffered formalin (10%, pH 7.4) for 24 hours, then processed using the conventional paraffin embedding technique [26]. Sections (5 μm thick) were sliced, mounted on slides deparaffinated in xylene, and rehydrated using decreasing ethanol concentrations. Slides were stained with hematoxylin and eosin (H&E) for routine histopathological setting. Stained sections were blindly evaluated using a light microscope (Leica, DM500) and photographed at magnification power 100 using a digital camera (EC3, Leica, Germany).

A histological lesion-scoring approach was adopted to show the severity of histopathological lesions. In each animal group, five H&E-stained slides (one slide/rat) were examined blindly to grade the pathological lesions. The severity of pathological lesions (glomerular and tubular injury scores, interstitial inflammation, interstitial fibrosis, and intratubular casts)was evaluated according to the percentage of tissue affected in the entire section as none (0): normal histology with zero involvement of the examined field, mild (1): 5–25% of the tested field was involved, moderate (2): 26–50% of the examined field was involved, severe (3): ˃50% of the examined field was involved. Each animal’s score was evaluated, and the median score was calculated for the various pathological alterations per group. The sums of the various lesion scores were used as the total renal tissue lesion score for each group [27].

### Statistical analysis

Data were analyzed using Prism software package version 5 (GraphPad Prism 5.0). The data were expressed as mean ± SD. The Kolmogorov-Smirnov test was used to study the normal distribution of the studied parameters. The analysis of variance (ANOVA) was made and followed by a post hoc (Bonferroni test) to compare the mean values between and within treated groups compared to untreated and control groups. For the histopathology scoring, nonparametric data were represented as median (min-max) and were analyzed by the Kruskal–Wallis Test, followed by Dunn’s post hoc test. Differences were considered statistically significant at *p*-value < 0.05. The correlation study was checked by Pearson’s correlation. The P value was assumed to be significant at *p* < 0.05.

## Results

### In silico phase

#### Prediction of the designed 3WJ-RNPs core (3WJ) and therapeutic 3WJ-RNPs (3WJ-Kapt/anti-miR-34a)

**Fig. 1:**
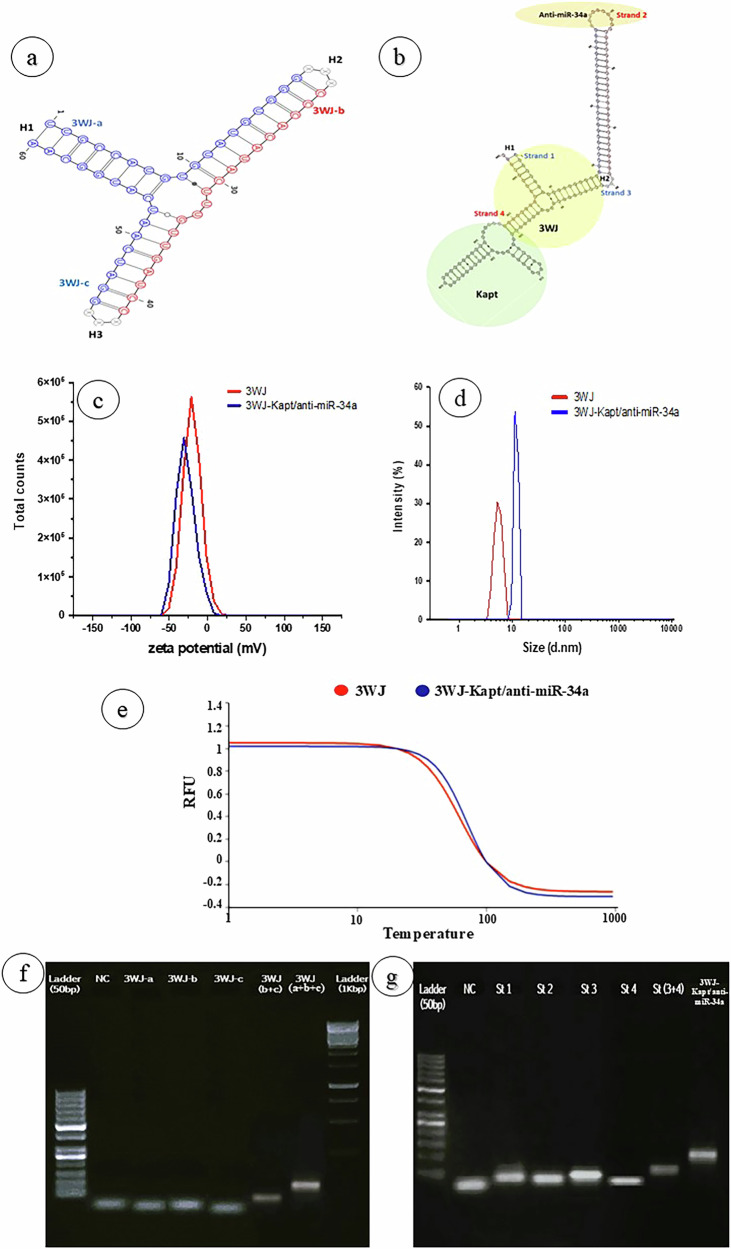
Prediction and characterization of the designed 3WJ-RNPs core (3WJ) and therapeutic 3WJ-RNPs (3WJKapt/anti-miR-34a). Folding results of the core three-way junction (3WJ) (**a**) and therapeutic three-way junction (3WJ-Kapt/anti-miR-34a) (**b**) using VfoldMCPX. 3WJ-a, 3WJ-b, and 3WJ-c; the basic three single-stranded RNA sequences, Strand 1; RNA sequence of 3WJ-a plus 3´-extending arm consisting of 25 ribonucleotides, Strand 2; Start from the 5´-end with anti-miR-34a hepta-deoxyribonucleotide (CACTGCC) followed by 25 Deoxyribonucleotides complementary to the extending arm of strand 1, Strand 3; RNA sequence of 3WJ-b plus 3´- extending 43 Deoxyribonucleotides of Kapt, Strand 4; RNA sequence of 3WJ-c. Characterization of RNA nanoparticles. Zeta potential of 3WJ, and 3WJ-Kapt/anti-miR-34a (**c**), Hydrodynamic diameter of 3WJ, and 3WJ-Kapt/anti-miR-34a (**d**) by dynamic light scattering (DLS). Melting curve of the prepared RNA-nanoparticles (**e**). Agarose gel electrophoresis of 3WJ with its single-stranded RNA sequences (**f**), and 3WJ-Kapt/anti-miR-34a with its individual four strands (st 1,2,3,4) against negative control (NC) (**g**) (no of replica=3).

The proper folding of these strands into correct 3WJ-RNP folding was predicted using the VfoldMCPX online tool (http://rna.physics.missouri.edu/vfoldMCPX2) which predicts the 2D structures of multi-strand RNA complexes. The folding results indicated the exact 3WJ-RNPs structure and indicated an exact 3WJ structure as illustrated in Fig. 1a with extending helix H2 (anti-miR-34a) and H3 (kidney targeting DNA aptamer (Kapt)) (Fig. 1b).

## In vitro phase: preparation and characterization of RNP

### Particle size (PS), and zeta potential (ZP) of RNPs

The ZP measures the electrical charge on the surface of RNP, and the results provide information regarding the long-term physical stability and aggregation tendency of RNP. Particles resist aggregation when ZP is sufficiently high (less than -30 mV or larger than +30 mV), which confers stability. The prepared RNP showed ZP of -17.7 ± 2.36 mV and -22.6 ± 0.17 mV for 3WJ and 3WJ-Kapt/anti-miR-34a, respectively (Fig. 1c) indicating the negatively charged property of RNA. The apparent hydrodynamic sizes of 3WJ and 3WJ-Kapt/anti-miR-34a are 5.279 ± 1.62 nm, and 13.06 ± 2.54 nm, respectively (Fig. 1d) as determined by dynamic light scattering (DLS).

### Assessment of melting temperatures (*Tm)* by thermal cycler

Characterization of the thermal stability of RNPs was done using real-time PCR. This method measures the Tm of nucleic acid samples by heating and cooling the samples. The nanoparticles undergo an initial denaturation phase when heated, followed by an assembly phase when they are cooled. The SYBR Green II fluorescence levels, which act as a reporter in the assembly of RNPs, were continuously monitored to get the melting curves (Fig. 1e). The Tm of each RNP was determined using nonlinear Sigmoidal fitting of the melting curves. It is evident from Fig. 1e that the therapeutic 3WJ’s Tm is 69.59 °C, while the core 3WJ’s Tm is 61.33 °C.

### Agarose gel electrophoresis

The electrophoresis of the negative control (NC), individual three ssRNAs of the core 3WJ (3WJ), the dimer of 3WJ-b + 3WJ-c, and the complete 3WJ trimer are shown in the plate (Fig. 1f). Only one major band was visualized from individual strands or 3WJ nanoparticles dimer and trimer. The band of the trimer of 3WJ has a higher molecular weight than the dimer of 3WJ-b + 3WJ-c, followed by the individual strands.

The electrophoresis of the negative control (NC), individual four strands (st 1,2,3,4) of the therapeutic 3WJ (3WJ-Kapt/anti-miR-34a), the dimer of strands 3 and 4, and the complete 3WJ-Kapt/anti-miR-34a tetramer (st 3 + 4 + 1 + 2) are shown in the plate (Fig. 1g). Only one major band was visualized from individual strands or 3WJ nanoparticles dimer and tetramer. The band of the tetramer of 3WJ-Kapt/anti-miR-34a has a higher molecular weight than the dimer of strands 3 and 4 followed by the individual strands. From Fig. 1g we can observe that the single strands of therapeutic 3WJ have higher molecular weights than that of the core strands except for the 3WJ-c and strand-4 because they are the same, while two strands of therapeutic RNPs (strand-1 and 3) are the 3WJ-a and b but extended by 25 nucleotides or Kapt sewunces, respectively. Consequently, the therapeutic 3WJ has a higher molecular size than the core 3WJ (171 vs. 54).

## In vivo phase

### Specific targeting of the kidney

**Fig. 2:**
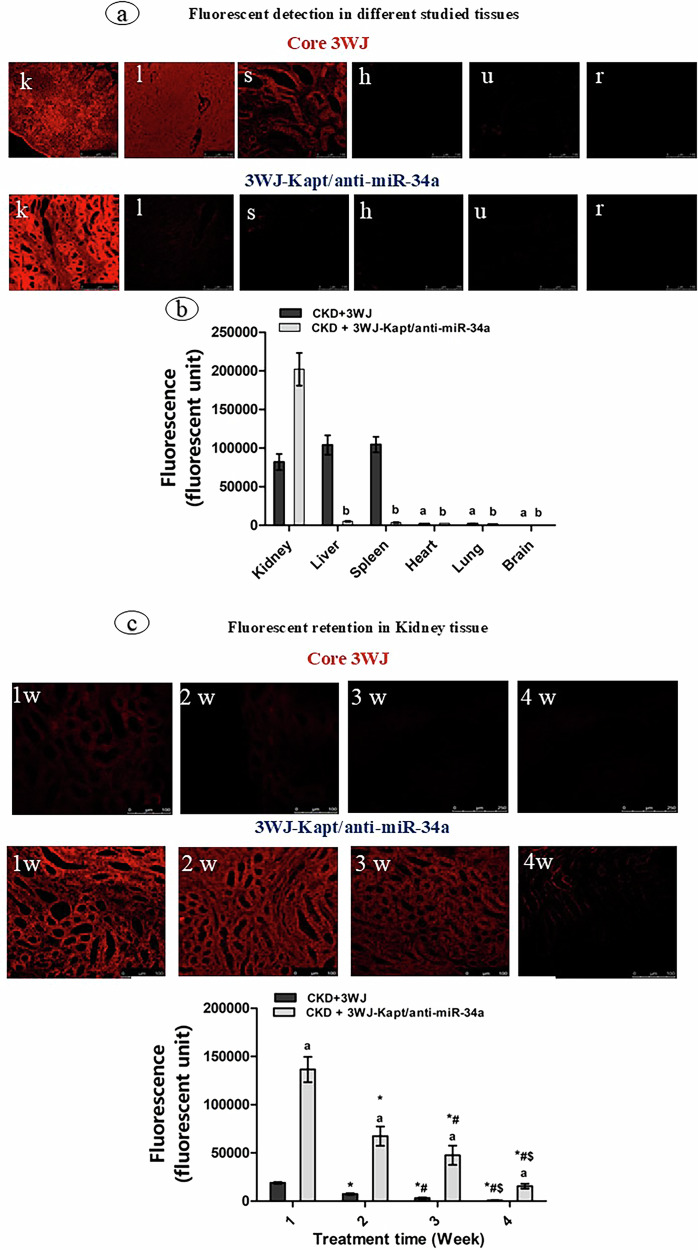
Tissues distribution of the core and therapeutic three-way junction. Fluorescent detection of the core three-way junction (3WJ) and therapeutic three-way junction (3WJ-Kapt/anti-miR-34a) (**a**) in the kidney (k), liver (l), spleen (s), heart (h), lung(u), and brain (r). The mean fluorescence values of 3WJ and 3WJ-Kapt/anti-miR-34a in the different organs (**b**). *Data are presented as mean ± SD. n* = *2. a: significantly different from the kidney of control mice + 3wj-c, b: significantly different from the kidney of control mice* + *3WJ-Kapt/anti-miR-34a by ANOVA followed by Bonferroni post hoc test (p* < *0.05)*. Fluorescent detection of the core three-way junction (3WJ) and therapeutic three-way junction (3WJ-Kapt/anti-miR-34a) in the kidney of chronic kidney disease (CKD) mice during 4 weeks of treatment (**c**). 1 week after treatment (1 W), 2 weeks after treatment (2 W), 3 weeks after treatment (3 W), 4 weeks after treatment (4 W). Fluorescent retention time (**d**), *Data are presented as mean ± SD. n* = *5. a: significantly different from CKD-3WJ mice by ANOVA followed by Bonferroni post hoc test (p* < 0.05) at the same time point.*: significantly different from the first week’s results, #: significantly different from the second week’s results, $ significantly different from the third week’s results by ANOVA followed by Bonferroni post hoc test (p < *0.05)*.

Fluorescence signals of Alexa-647 from the 3WJ or 3WJ-Kapt/anti-miR-34a were detected by examining microsections of the dissected organs (kidney, liver, spleen, heart, lung, and brain) using a confocal microscope (LEICA DMI8) with an excitation at 640 nm and emission at 660 nm for a 1 min exposure. The untargeted 3WJ showed unspecific distribution into different tissues, especially to the kidney, liver, and spleen, with low levels in the heart and lungs and with no detection in the brain. The targeted 3WJ-Kapt/anti-miR-34a using kidney aptamer (Kapt) showed specific targeting to the kidney tissues with very low detection levels in the liver and spleen and no detection level in other tissues (Fig. 2a, b).

### Toxicity experiment

The treatment of healthy mice with a single intravenous injection with any of the prepared RNPs (3WJ or 3WJ-Kapt/anti-miR-34a) for 4 weeks didn’t significantly affect any of the weight, the kidney function tests (serum urea and creatinine levels), or liver function tests (ALT and AST activities). Serum and kidney levels of KIM-1 and NAG weren’t significantly affected, in addition to hemoglobin, EPO, and MDA. Control mice injected with 3WJ showed a significant increase in the renal level of TNF-α with a non-significant change in IL-6, while mice injected with 3WJ-Kapt/anti-miR-34a showed non-significant changes in both TNF-α and IL-6 (Table 2), confirming the safety of the prepared RNPs.

**Table 2:** Toxicity study parameters in serum and kidney of control mice injected with core three-way junction (3WJ) or therapeutic three-way junction (3WJ-Kapt/anti-miR-34a).

Groups	Control	Control + 3WJ	Control + 3WJ-Kapt/anti-miR-34a
Parameter			
Weight (gm)	35.60 ± 0.89	34.00 ± 1.22	35.40 ± 1.14
ALT (U/L)	47.9 ± 7.33	48.30 ± 6.04	53.40 ± 7.44
AST (U/L)	148.3 ± 8.93	139.80 ± 7.26	154.40 ± 8.41
Urea (mg/dl)	34.4 ± 6.35	31.92 ± 3.01	35.20 ± 4.55
Creatinine (mg/dl)	0.37 ± 0.04	0.33 ± 0.07	0.41 ± 0.07
KIM-1 Serum (pg/ml)	61.86 ± 15.00	67.44 ± 12.89	67.96 ± 12.94
KIM-1 Kidney (pg/mg protein)	3.23 ± 0.52	3.43 ± 0.32	3.46 ± 0.61
NAG Serum (ng/ml)	28.68 ± 4.15	27.22 ± 3.71	32.24 ± 5.01
Hemoglobin (g/dl)	12.51 ± 0.86	12.92 ± 0.81	11.64 ± 0.93
EPO (pg/ml)	103.58 ± 8.29	98.07 ± 8.95	101.00 ± 7.55
MDA (nmol/g tissue)	21.86 ± 4.02	25.20 ± 2.66	26.56 ± 3.86
TNF-α (pg/ mg protein)	3.39 ± 0.64	6.47 ± 0.98^a^	3.87 ± 0.58^b^
IL-6 (pg/ mg protein)	5.81 ± 2.16	7.32 ± 0.96	6.48 ± 1.56

Data presented as mean ± SD. *n *= 5.

*ALT* alanine aminotransferase, *AST* aspartate aminotransferase, *KIM-1* Kidney injury molecule-1, *NAG* N-acetyl-β-D-glucosaminidase, *EPO* Erythropoietin, *MDA* malondialdehyde, *TNF-α* Tumor necrosis factor-α, *IL-6* Interleukin-6.

^a^Significantly different from control mice.

^b^Significantly different from control mice treated with 3WJ by ANOVA followed by Bonferroni post hoc test (*p* < 0.05).

## Treatment of CKD mice

### Retention of the 3WJ and the 3WJ-Kapt/anti-miR-34a within the kidney tissues of treated mice

The confocal microscope detected the fluorescence of the Alexa467 in sections of the kidney tissues of CKD during the 4-week treatment period. The 3WJ and the 3WJ-Kapt/anti-miR-34a showed a duration-dependent decline in fluorescence activity. However, the 3WJ-Kapt/anti-miR-34a was more concentrated in the kidney tissues than the 3WJ throughout the experimental period. The fluorescence in the CKD-3WJ-treated mice showed almost no detectable fluorescence in the 4^th^ week, while CKD-3WJ-Kapt/anti-miR-34a showed considerable retention in the kidney tissues (Fig. 2c, d).

### Kidney and liver function tests and animal weights

**Fig. 3:**
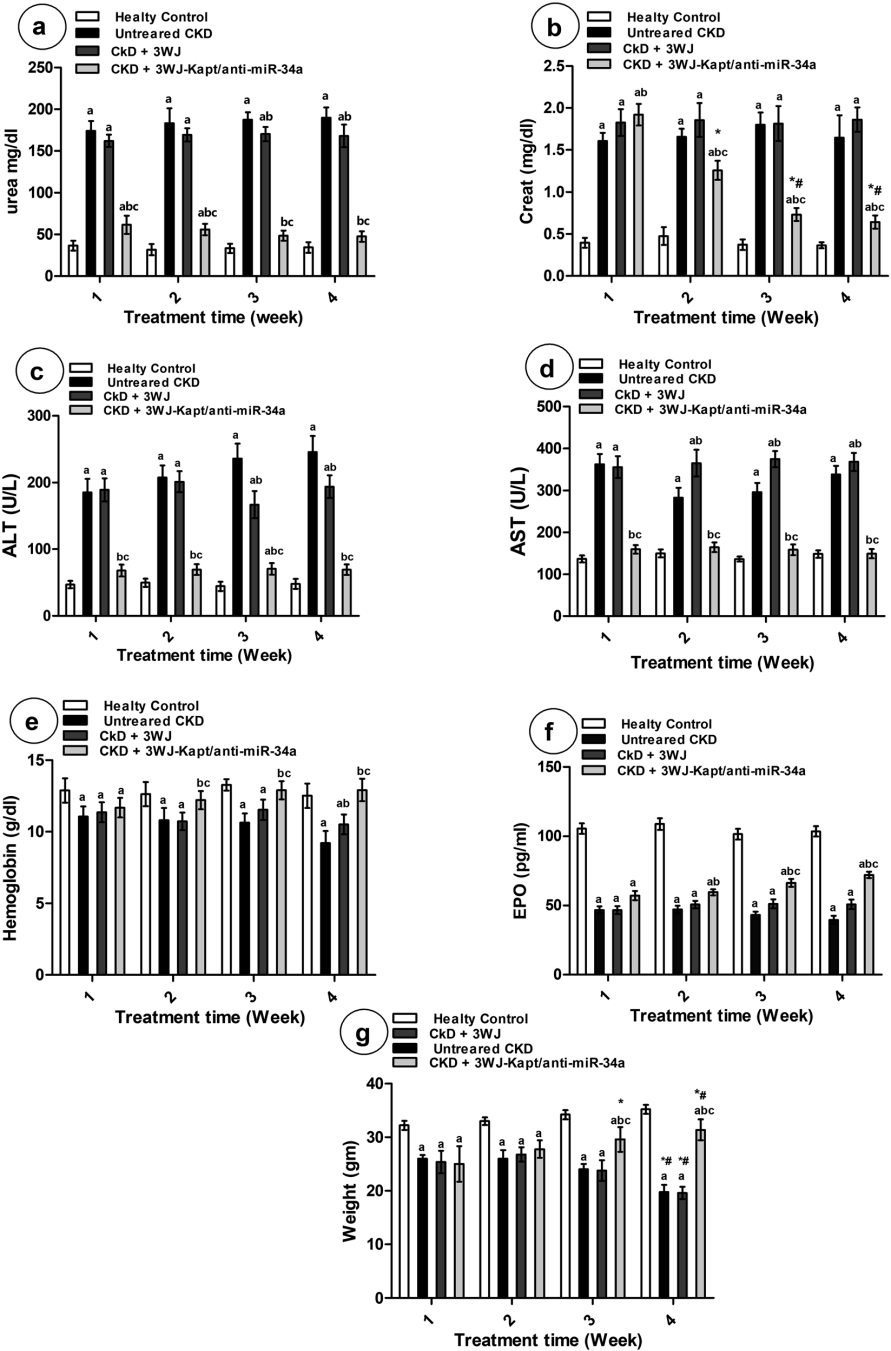
Kidney and liver function tests and animal weights of different experimental groups at different experimental periods. Kidney function tests (**a**, **b**), liver function tests (**c**, **d**), hemoglobin (**e**), EPO (**f**), weight (**g**) Data are presented as mean ± SD. *n *= 5. **a** significantly different from control mice, **b** significantly different from untreated CKD mice, **c** significantly different from CKD-3WJ mice by ANOVA followed by Bonferroni post hoc test (*p *< 0.05) at the same time point. *: significantly different from the first week’s results, #: significantly different from the second week’s results by ANOVA followed by Bonferroni post hoc test (*p *< 0.05). ALT alanine aminotransferase, AST aspartate aminotransferase, EPO Erythropoietin.

The serum urea, creatinine, ALT, and AST activities showed significantly higher levels in CKD untreated mice throughout the 4-week experimental period compared to the control mice. It was observed that treatment of CKD mice with 3WJ didn’t significantly affect the serum urea, creatinine, and ALT activity in the 1^st^ and 2^nd^ weeks, while the 3^rd^ and 4^th^ weeks showed a significant reduction in ALT activity and a slight but significant decline in urea levels compared to the untreated mice, while elevated AST activity compared to the untreated mice was observed from the 2^nd^ week and thereafter. Treatment with the 3WJ-Kapt/anti-miR-34a significantly reduced ALT and AST activities compared to the untreated mice and completely normalized their levels as early as the 1^st^ week of treatment. While urea levels also showed a significant and marked decline starting from the 1^st^ week of treatment and thereafter, creatinine levels started from the 2^nd^ week of treatment to show a prompt decrease (Fig. 3a–d). The final weight showed a significant reduction in untreated CKD mice throughout the 4-week experimental period compared to the control mice. It was observed that treatment of CKD mice with 3WJ didn’t significantly affect weight compared to untreated mice. Treatment with the 3WJ-Kapt/anti-miR-34a didn’t significantly affect weight in the first and second week while the third and fourth week of treatment showed a prompt increase compared to untreated mice (Fig. 3g).

### Renal injury markers

**Fig. 4:**
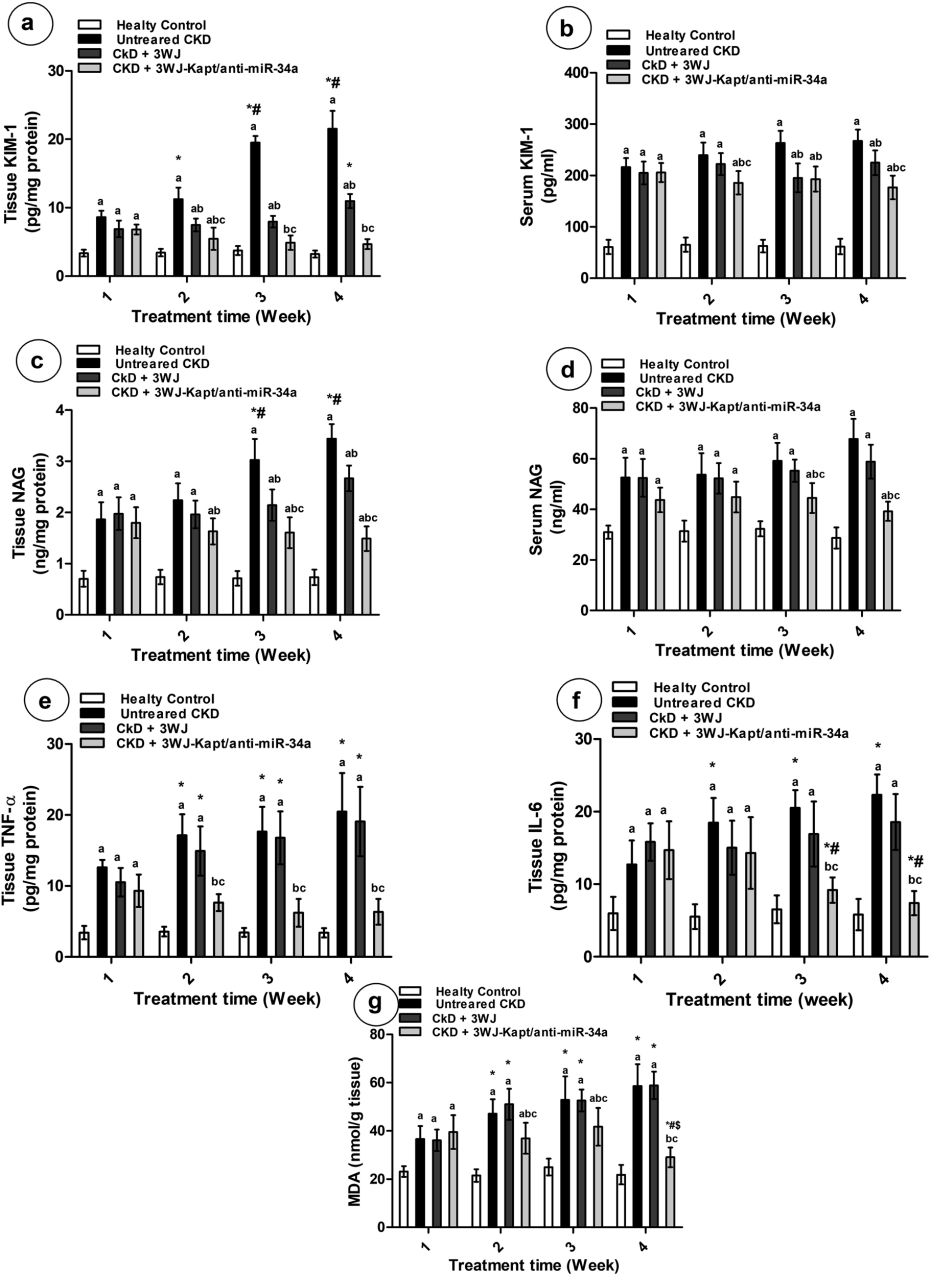
Kidney injury, inflammatory, and oxidative stress markers different experimental groups at different experimental periods. Kidney injury markers in serum and tissues (**a**–**d**), inflammatory markers (**e**, **f**), oxidative stress marker (**g**). Data are presented as mean ± SD. *n *= 5. **a** significantly different from control mice, **b** significantly different from untreated CKD mice, **c** significantly different from CKD-3WJ mice by ANOVA followed by Bonferroni post hoc test (*p *< 0.05) at the same time point. *Significantly different from the first week’s results, #significantly different from the second week’s results by ANOVA followed by Bonferroni post hoc test (*p *< 0.05). KIM-1 Kidney injury molecule-1, NAG N-acetyl-β-D-glucosaminidase, EPO Erythropoietin, TNF-α Tumor necrosis factor-α, IL-6 Interleukin-6, MDA malondialdehyde.

The renal and serum KIM-1 and NAG levels were significantly elevated in CKD untreated mice throughout the 4-week experimental period compared to the control mice (Fig. 4a–d), while serum hemoglobin and EPO revealed decreased levels (Fig. 3e, f). Treatment of CKD mice with 3WJ slightly reduced serum and renal KIM-1 levels compared to the untreated mice starting from the 2nd week. However, serum NAG didn’t significantly change from untreated mice but showed a significant decrease in renal NAG from 3rd week. The hemoglobin and EPO levels didn’t significantly change except for the 4th week which showed a slight increase in the hemoglobin level compared to untreated mice. Treatment with the 3WJ-Kapt/anti-miR-34a significantly elevated serum hemoglobin and EPO levels and declined serum and renal KIM-1, as well as renal NAG from the 2nd week of treatment compared to the untreated mice. In the 3rd week, serum NAG started to decline, where renal KIM-1 was able to normalize its level.

### Renal inflammatory and oxidative stress markers

The renal TNF-α, IL-6, and MDA were significantly elevated in CKD untreated mice throughout the 4-week experimental period compared to the control mice. While treatment of CKD mice with 3WJ didn’t significantly affect renal TNF-α, IL-6, and MDA compared to the untreated mice, treatment with the 3WJ-Kapt/anti-miR-34a showed a significant decline and completely normalized the renal TNF-α IL-6, and MDA levels compared to the untreated mice from the 2nd, 3rd and 4th week of treatment, respectively (Fig. 4e–g).

## Gene expression analysis

### Renal expression of miR-34a

**Fig. 5:**
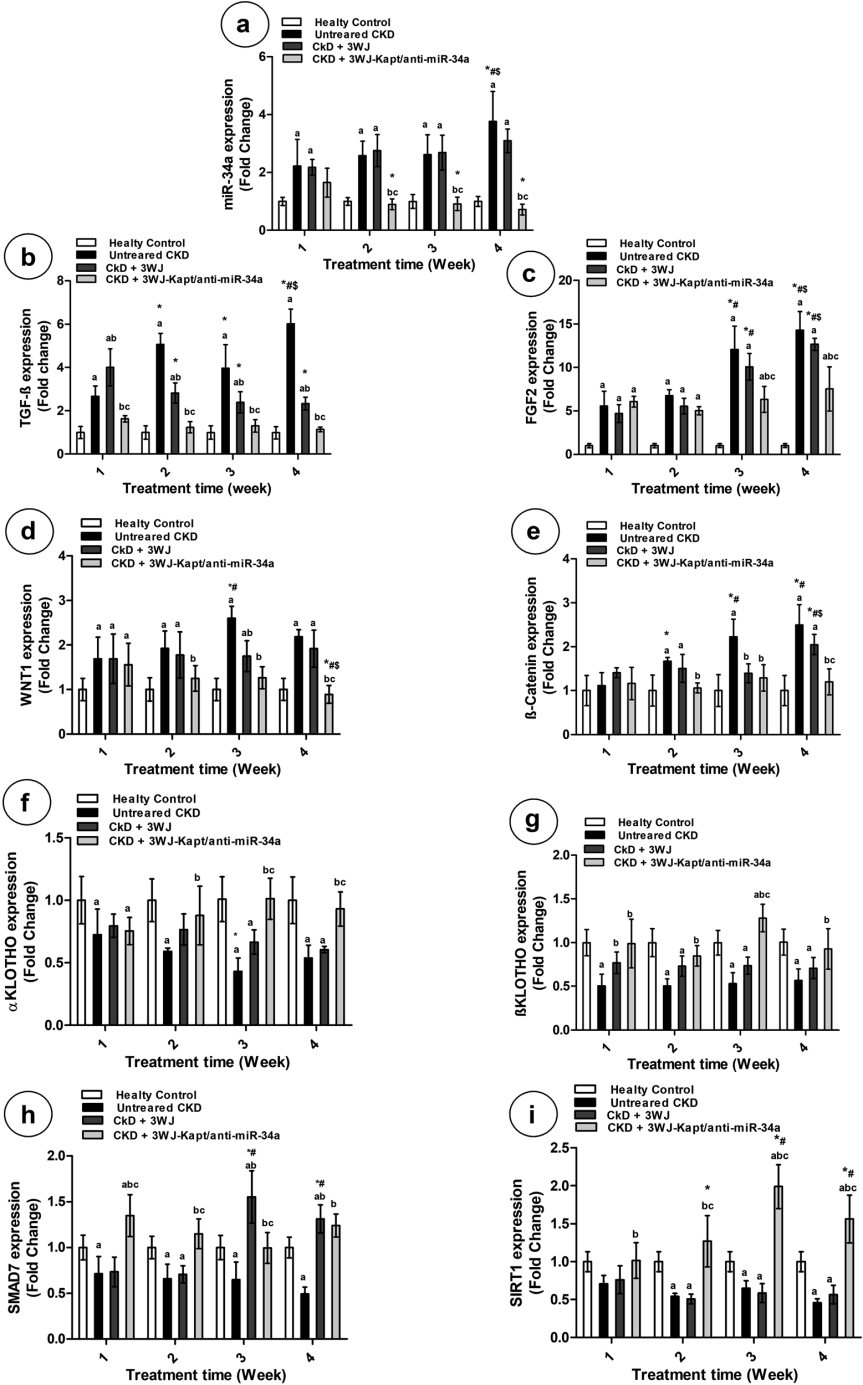
Results of renal mRNA gene expression of different experimental groups at different experimental periods. Renal miR-34a expression (**a**), renal fibrotic gene expressions (**b**–**e**). Renal anti-fibrotic gene expressions (**f**–**i**). Data are presented as mean ± SD. *n* = 5. **a** Significantly different from control mice, **b** Significantly different from untreated CKD mice, **c** Significantly different from CKD-3WJ mice by ANOVA followed by Bonferroni post hoc test (*p* < 0.05) at the same time point. *Significantly different from the first week’s results, #significantly different from the second week’s results by ANOVA followed by Bonferroni post hoc test (*p* < 0.05). TGF-β Transforming growth factor-β, FGF2 Fibroblast Growth Factor 2, WNT1 Wingless-related integration site, Smad7 Suppressor of Mothers against Decapentaplegic, SIRT1 Sirtuin-1.

The expression of renal miR-34a showed time-dependent induction in the untreated CKD mice compared to the control mice. Treatment of CKD mice with 3WJ showed non-significant changes in miR-34a expression throughout the 4 weeks of treatment compared to untreated mice. However, the 3WJ-Kapt/anti-miR-34a treatment was able to normalize renal miR-34a expression starting from the 2^nd^ week of treatment (Fig. 5a).

### Renal fibrotic markers

**Fig. 6:**
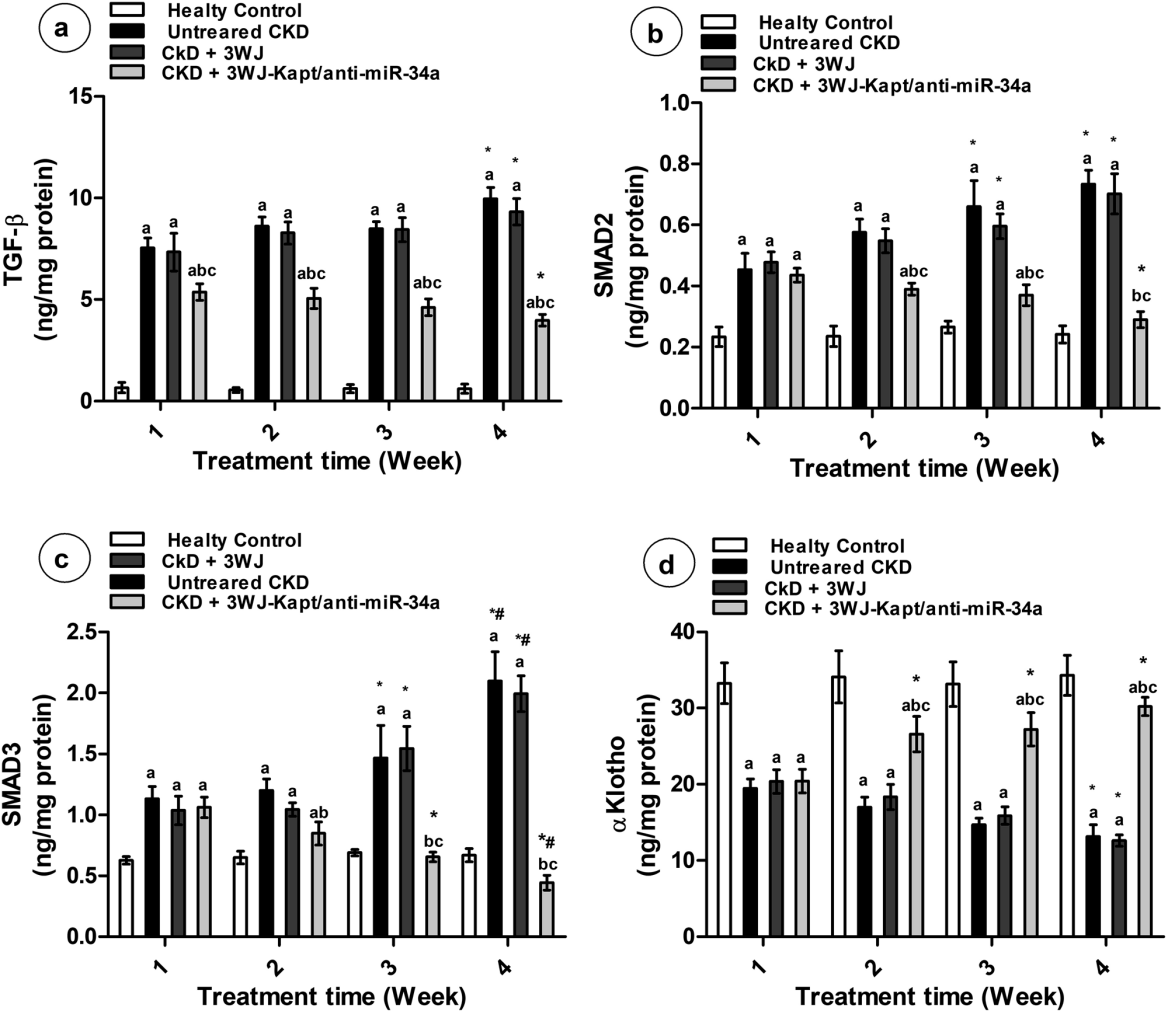
Results of renal protein contents of different experimental groups at different experimental periods. Renal protein content of TGF-β (**a**), SMAD2 (**b**), SMAD3 (**c**), and Klotho (**d**). Data are presented as mean ± SD. *n* = 5. **a** Significantly different from control mice, **b** significantly different from untreated CKD mice, **c** significantly different from CKD-3WJ mice by ANOVA followed by Bonferroni post hoc test (*p* < 0.05) at the same time point. *Significantly different from the first week’s results, #Significantly different from the second week’s results by ANOVA followed by Bonferroni post hoc test (*p* < 0.05). Smad Suppressor of Mothers against Decapentaplegic.

The renal expression of TGF-β, FGF2, WNT1, and β-catenin showed time-dependent induction in the untreated CKD mice compared to the control mice (Fig. 5b–e). Similarly, the renal protein contents of TGF-β, SMAD2, and SMAD3 showed marked time-dependent elevation in untreated mice with CKD (Fig. 6a–c). The treatment of CKD mice with the core 3WJ showed a significant decline in TGF-β expression starting from 2^nd^ week and in WNT1 and β-catenin expressions starting from the 3^rd^ week compared to untreated mice, where the FGF2 expression was not affected, also the protein contents of TGF-β, SMAD2, and SMAD3 are not affected by the 3WJ treatment (Figs. 5b–e, 6a–c). The CKD-3WJ-Kapt/anti-miR-34a mice showed complete normalization of the renal TGF-β expression starting from the 1^st^ week of treatment, while it was able to normalize the β-catenin and WNT1 expression starting from the 2^nd^ week. However, the expression level of renal FGF2 was only affected starting from the 3^rd^ week of treatment (Fig. 5b–e). At the protein level, the CKD mice treated with 3WJ-Kapt/anti-miR-34a showed a significant time-dependent decline in both SMAD2 and SMAD3 which became significant starting from the 2^nd^ week of treatment and completely normalized after 4 weeks. On the other hand, the renal content of TGF-β showed a significant decline upon treatment with 3WJ-Kapt/anti-miR-34a starting from the 2^nd^ week but its level didn’t show further decline with time (Fig. 6a–c).

### Renal anti-fibrotic markers

The renal expression of α Klotho, β Klotho, SMAD7, and SIRT1 in the untreated CKD mice showed significant downregulation compared to the control mice in a time-dependent manner. While the treatment of CKD mice with the 3WJ didn’t affect the gene expression of all parameters except SMAD7 from the 3^rd^ week, the CKD-3WJ-Kapt/anti-miR-34a mice showed complete normalization of all parameters from the 1^st^ and 2^nd^ weeks. Interestingly, SMAD7, SIRT1, and β Klotho expression were even higher than the control mice on the 3^rd^ and 4^th^ weeks (Fig. 5f–i). At the protein level, the renal content of Klotho in the untreated CKD mice showed a significant decrease compared to the control mice. The treatment of CKD mice with the 3WJ didn’t affect the α Klotho level. The CKD-3WJ-Kapt/anti-miR-34a mice showed a significant increase in α Klotho content compared to untreated mice in a time-dependent manner (Fig. 6d).

### Histopathological examination

**Fig. 7:**
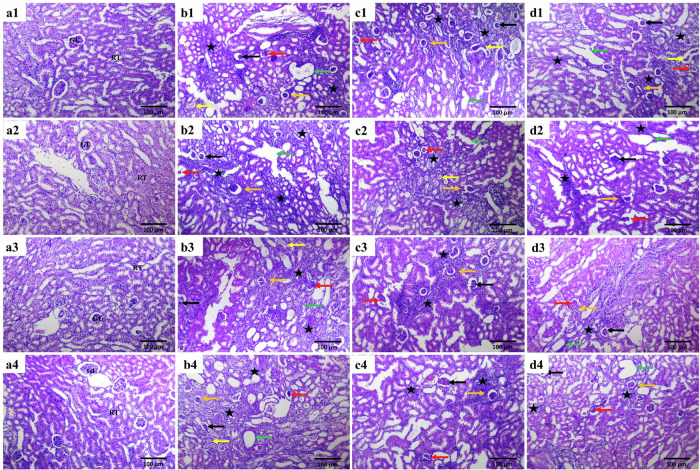
Histological analysis. Representative photomicrographs of mice kidneys from different experimental groups at different experimental periods (H&E stain, x100), (**a1**–**a4**) mice from the control group, (**b1–b4**) mice from the CKD group, (**c1–c4**) mice from the 3WJ group and (**d1**–**d4**) mice from the 3WJ-Kapt/anti-miR-34a group. **a1**, **b1**, **c1**, **d1** one week, **a2**, **b2**, **c2**, **d2**: two weeks, **a3**, **b3**, **c3**, **d3**: three weeks and **a4**, **b4**, **c4**, **d4**: four weeks treatments. [Normal renal glomeruli (GL), renal tubule (RT), dilatation of the renal tubular lumen (green arrow), fibrous tissue proliferation and interstitial mononuclear inflammatory cell infiltrations (star), atrophied glomeruli (black arrow), necrotic glomeruli (red arrow), periglomerular fibrosis (orange arrow) and intratubular cast formation (yellow arrow)].

The kidneys of the control groups revealed normal histological morphology of the renal parenchyma with well-defined glomeruli and tubules throughout the experimental period (Fig. 7a1–a4). Examined renal tubules of CKD mice showed signs of tubular damage, including dilatation of the tubular lumen, disorganized tubular arrangement, cytoplasmic lysis or swelling of the epithelial cell nucleus, tubular epithelium attenuation and necrosis, and desquamation of epithelial cells. The tubular lumen contains dark eosinophilic necrotic debris and hyaline casts. The renal parenchyma showed tubulointerstitial nephritis with mononuclear cell infiltrations, interstitial inflammation, and interstitial fibrosis. The CKD mice also recorded glomerular lesions, including glomerular atrophy and necrosis, associated with distension of the Bowman’s space and periglomerular fibrosis. The severity of pathological lesions was time-dependent (Fig. 7b1–b4), showing an increased median score over the weeks.

The CKD mice treated with the CKD-3WJ showed slightly ameliorated pathological lesions compared to the untreated mice during the four weeks (Fig. 7c1–c4). 3WJ-Kapt/anti-miR-34a treatment ameliorated the above-mentioned pathological lesions and inhibited the increase in tubular damage scores in a time-dependent manner, and this effect was most robust in mice treated with 3WJ-Kapt/anti-miR-34a at the fourth week (Fig. 7d1–d4). The median of the sum of histological lesion scores is presented in Table 3.

**Table 3:** Histological lesion scores in CKD mice untreated, core three-way junction (3WJ) or therapeutic three-way junction (3WJ-Kapt/anti-miR-34a) treated.

Groups	Control	Untreated CKD	CKD + 3WJ	CKD + 3WJ-Kapt/anti-miR-34a
Time				
**Glomerular injury**				
**Week 1**	0.00 (0–0)	3 (2-3) ^a^	2 (2-3) ^a^	2 (2-3)^a^
**Week 2**	0.00 (0–0)	3 (2-3) ^a^	2 (2-3) ^a^	2 (1-2)^a,b,c,d^
**Week 3**	0.00 (0–0)	3 (2-3) ^a^	2 (2-3) ^a^	1 (1-2) ^a,b,c,d,e^
**Week 4**	0.00 (0–0)	3 (2-3) ^a^	2 (2-3) ^a^	1 (1-2) ^a,b,c,d,e^
**Tubular injury**				
**Week 1**	0.00 (0–0)	3 (2-3) ^a^	2 (2-3) ^a^	2 (1-2) ^a^
**Week 2**	0.00 (0–0)	3 (2-3) ^a^	2 (1-3) ^a^	2 (2-3) ^a,b,c,d^
**Week 3**	0.00 (0–0)	3 (2-3) ^a^	2 (2-3) ^a^	1 (1-2) ^a,b,c,d,e^
**Week 4**	0.00 (0–0)	3 (2-3) ^a^	3 (2-3) ^a^	1 (1-2) ^a,b,c,d,e^
**Interstitial infilammation**				
**Week 1**	0.00 (0–0)	3 (2-3) ^a^	2 (2-3) ^a^	3 (2-3) ^a^
**Week 2**	0.00 (0–0)	3 (2-3) ^a^	3 (2-3) ^a^	2 (2-3) ^a,b,c,d^
**Week 3**	0.00 (0–0)	3 (3-3) ^a^	3 (2-3) ^a^	2 (1-2) ^a,b,c,d^
**Week 4**	0.00 (0–0)	3 (3-3) ^a^	3 (2-3) ^a^	1 (1-2) ^a,b,c,d,e^
**Interstitial fibrosis**				
**Week 1**	0.00 (0–0)	1 (0–1) ^a^	1 (0–1) ^a^	1 (0–1) ^a^
**Week 2**	0.00 (0–0)	1 (0–1) ^a^	1 (0–1) ^a^	1 (0–1) ^a^
**Week 3**	0.00 (0–0)	1 (0–2) ^a^	1 (0–2) ^a^	1 (0–2) ^a,b,c,d,e^
**Week 4**	0.00 (0–0)	2 (0–3) ^a^	2 (1–3) ^a^	1 (0–1) ^a,b,c,d,e^
**Intratubular casts**				
**Week 1**	0.00 (0–0)	3 (2–3) ^a^	2 (1–3) ^a^	3 (2–3) ^a^
**Week 2**	0.00 (0–0)	3 (2–3) ^a^	2 (2–3) ^a^	2 (1–3) ^a,b,c,d^
**Week 3**	0.00 (0–0)	3 (2–3) ^a^	2 (2–3) ^a^	2 (1–2) ^a,b,c,d^
**Week 4**	0.00 (0–0)	3 (2–3) ^a^	3 (2–3) ^a^	1 (1–2) ^a,b,c,d,e^
**Total lesion score**				
**Week 1**	0.00 (0–0)	11 (9–12) ^a^	10 (7–11) ^a^	9 (7–11) ^a^
**Week 2**	0.00 (0–0)	12 (10–12) ^a^	11 (8–12) ^a^	8 (6–9) ^a,b,c,d^
**Week 3**	0.00 (0–0)	12 (10–14) ^a^	11 (9–14) ^a^	7 (5–8) ^a,b,c,d,e^
**Week 4**	0.00 (0–0)	13 (12–14) ^a^	12 (9–12) ^a^	7 (6–8) ^a,b,c,d,e^

Data are presented as median (min–max). *n *= 5.

^a^Significantly different from control mice.

^b^Significantly different from untreated CKD mice.

^c^Significantly different from CKD-3WJ mice.

^d^Significantly different from the first week’s results.

^e^Significantly different from the second week’s results by Kruskal–Wallis test that was used to analyze the renal tissue injury score, followed by Dunn’s post-hoc test (*p *< 0.05).

### Correlation studies

Table 3 in the supplementary file shows correlation studies between different parameters in untreated CKD, CKD-3WJ, and CKD-3WJ-Kapt/anti-miR-34a. Our study’s data revealed that miR-34a positively correlated with the expression of profibrotic genes (TGF-β, FGF2, WNT1, β-Catenin), profibrotic protein content (SMAD2, SMAD3), and inflammatory markers (TNF-α, IL-6) while it negatively correlated with antifibrotic genes (SMAD7, SIRT1, αklotho, βklotho). Moreover, miR-34a was positively correlated with serum and tissue kidney injury markers such as KIM-1 and NAG.

## Discussion

For the first time, the present study reported the promising therapeutic targeting of RNA nanoparticles harboring LNA anti-miR-34a (3WJ-Kapt/anti-miR-34a) against CKD progression in mice. Through suppressing the miR-34a pathway in the kidney tissues, the prepared 3WJ-Kapt/anti-miR-34a significantly ameliorated the main pathways involved in the pathogenesis of CKD and renal fibrosis.

In the current study, we designed a therapeutic RNA particle against CKD based on the 3WJ-RNPs which have two functionalized domains; the first domain is the extending arm of 25 ribonucleotides ended with a single anti-miR-34a hepta-deoxyribonucleotide (CACTGCC) as a locked nucleic acid (LNA) was attached to the H2 helix of the 3WJ. The second domain is 43 nucleotides DNA-aptamer targeted for kidney cells (Kapt) was attached to the H3 helix.

Aptamers are single-stranded nucleic acid molecules that can fold into complex three-dimensional structures, forming binding pockets and clefts for the specifi c recognition and tight binding of molecular targets. Aptamers have all of the advantages of antibodies, besides having unique thermal stability, ease of synthesis, reversibility, and little immunogenicity [28]. The work of Ranches et al. was used as the source of the kidney-targeted aptamer (Kapt) sequence utilized in this study [21]. They identified new cell-internalizing DNA aptamers with high specificity to renal proximal tubular epithelial cells (RTEC) using cell systematic evolution of ligands by exponential enrichment (cell-SELEX). This aptamer harbors G-rich or G-quartet motifs that have a high propensity to form G-quadruplexes which are implicated in different biological processes (e.g., DNA replication, gene expression, and telomerase maintenance). The cell surface markers like megalin and cubilin receptors may serve as target recognition sites of the DNA aptamer that is being internalized via clathrin-mediated endocytosis [21].

The proper folding of the designed 3WJ-Kapt/anti-miR-34a into the 3WJ configuration was predicted using the VfoldMCPX online tool and confirmed practically using gel electrophoresis which confirmed the binding of the 3 stands of core and the four strands of therapeutic RNPs. However, the proper formation of the 3-WJ configuration may need to be confirmed using other techniques like atomic force microscopy (AFM) or cryo-electron microscopy which is considered as one limitation of our study. Detecting the fluorescence signals of Alexa-647 fluorophore attached to the 3WJ-c strand in the different sections of kidney tissues and other organs revealed that the targeted 3WJ-Kapt/anti-miR-34a using Kapt can specifically target the kidney tissues with no or little accumulation in the other organs. On the contrary, the untargeted 3WJ showed unspecific distribution into different tissues, especially to the kidney, liver, and spleen, with low levels in the heart and lungs and with no detection in the brain.

The shape and size of RNPs have a significant effect on their in vivo behavior including organ accumulation and time of circulation [11]. Dynamic light scattering (DLS) and gel electrophoresis are considered the most common methods to measure the hydrodynamic diameter and demonstrate the relative size of RNPs. The sizes and zeta potentials of the prepared 3WJ and 3WJ-Kapt/anti-miR-34a were 5.279 ± 1.62 nm, and 13.06 ± 2.54 nm, and -17.7 ± 2.36 mV and -22.6 ± 0.17 mV, respectively. These data are in accordance with the previous studies which reported the size and zeta-potential of RNA nanoparticles constructed from multiple 3WJs [29–31]. These sizes are sufficiently large to avoid rapid excretion by kidneys, but sufficiently small to enter target cells via receptor-mediated endocytosis [11]. Also, the zeta potentials confers stability because particles resist aggregation. Gel electrophoresis results indicated a band of the high molecular weight assembled 3WJ complexes (trimer of 3WJ and tetramer of 3WJ-Kapt/anti-miR-34a), which have higher molecular weight than the dimers and the individual strands.

Besides size and shape, the enzymatic and thermal stability of RNPs may also be modified by altering the structure and composition of the composed RNA strands. The most often utilized modifications for creating thermally stable RNPs are 2’-Fluoro (2′-F) ribonucleotides (C and U) that contain a fluorine molecule at the 2’ ribose position (instead of a 2’-hydroxyl group in an RNA monomer) and LNA. The primary advantages of these changes are enhanced nuclease resistance, melting temperature (Tm), and binding affinity [32]. The data obtained from our results confirmed this finding, as the Tm of the 3WJ is 61.33 °C, while that for the 3WJ-Kapt/anti-miR-34a is 69.59 °C, indicating extra-thermal stability of these modified RNPs.

The confirmed effective targeting of the constructed 3WJ-Kapt/anti-miR-34a in the current study has been associated with the safety of both nanoparticles (3WJ and 3WJ-Kapt/anti-miR-34a) in normal mice after 4 weeks of systemic injection. The non-protein nature of 3WJ-RNPs allows them to pass through the glomerulus and overcome the filtration size limit, displaying favorable biodistribution profiles and pharmacokinetics in vivo with no or low toxicity [11]. Those properties together with the confirmed safety profile encouraged us to explore the therapeutic potential of the properly designed and constructed multi-functional (Kapt and anti-miR-34a) 3WJ-RNPs in mice models of CKD, being of complex nature, categorized with high mortality and morbidity rate [33] with no available safe and effective treatments for renal fibrosis [34].

The adenine-induced CKD mice in the current study developed the classical picture of CKD including marked elevation in urea, creatinine, a time-dependent increase in serum and tissue KIM-1, and NAG, oxidative stress, and inflammatory markers, besides the elevated liver enzyme activities along the experimental period. The elevated liver enzymes in mice with adenine-induced CKD may result from the direct hepatotoxic effects of adenine and/or uremic toxin accumulation [35]. Also, CKD mice have significantly declined hemoglobin levels and EPO levels. Since KIM-1 overexpression promotes macrophage chemotaxis, which further induces fibrosis and renal tubular inflammation, KIM-1 is regarded as a sensitive biomarker for CKD [36]. Moreover, NAG is the brush border enzyme produced in proximal tubule cells; its overexpression implies tubular injury [37].

Such results were confirmed upon histopathology examination showing typical tubular damage [38] characterized by tubular atrophy, interstitial fibrosis, and glomerulosclerosis. These findings confer impaired kidney function and the reliability of the chosen model in causing crystallization in the proximal tubular epithelia followed by inflammation and subsequent fibrosis [23]. However, the application of a more specific stain for fibrosis as the Masson trichrome stain may provide more information about the severity of fibrosis and connective tissue deposition. Besides, the common CKD-associated renal anemia is due to iron deficiency, chronic inflammation, shortened erythrocyte half-life, and, most significantly EPO deficiency [6].

The effective targeting of 3WJ-Kapt/anti-miR-34a into the kidney tissue has been confirmed in the present study, where it showed pronounced accumulation in the kidney tissues throughout the experimental period (4 weeks), While it showed a slight duration-dependent decline, the kidney retained a considerable fluorescence after 4 weeks of injection, indicating sequestering of the 3WJ-Kapt/anti-miR-34a in the target tissues. The persistence of RNA nanoparticles in tissues for a long time depends on their structural stability, resistance to degradation, and clearance mechanisms. While 3WJ RNA nanoparticles are designed to be stable and resistant to enzymatic degradation, their long-term persistence in tissues is generally limited due to their biodegradability and clearance by the body’s natural processes and most studies demonstrated the accumulation and persistence of the RNA nanoparticles within 72 hours post-injection [10].

The prolonged therapeutic effect (functionality) of RNPs 3WJ-Kapt/anti-miR-34a may be explained by using LNA and 2F-modified ribonucleotides also it may be the miR34 knockdown affects downstream pathways and becomes functional along the experimental period. The nucleotide modifications increase the binding affinity and stability of RNA, potentially extending its functional duration in vivo. The half-life of these RNP in circulation was documented to be approximately 24 hours and the RNPs are detectable in the blood for up to 48 hours, indicating a prolonged presence compared to unmodified RNA, which is cleared within 2 hour [39]. While the half-life gives an idea about the stability of RNPs in circulation, their function means the period during which the nanoparticles remain intact and capable of performing their intended therapeutic role intracellularly. Given their stability and targeted delivery to renal cells, it seems they remain functional for a longer time than the half-life depending on the threshold for effectiveness. The observed long-lasting detection of Alexa-647 signals in the kidney tissues 4 weeks post-injection may be explained by using LNA and 2F-modified strands that confer thermodynamic stability. However, also it was possible that Alexa-647 may be cleaved off the 3WJ and retained in the kidney tissue as the stability of Alexa-647 inside cells is generally excellent, making it a widely used fluorophore for intracellular imaging and tracking. Its stability is attributed to its photostability, resistance to photobleaching, and chemical robustness in the intracellular environment [40]. So, tracing the intracellular stability and kinetic of RNPs using radiolabeled strands requires further investigation.

The 4^th^ week of treatment showed the best amelioration effect in CKD mice, where 3WJ-Kapt/anti-miR-34a ameliorated the disrupted urea, creatinine, hemoglobin, and EPO levels. Histologically, it also ameliorated the observed pathological lesions, restored renal cell architectures, and inhibited the increase in tubular damage scores in a time-dependent manner. These improvements were associated with significantly decreased levels of serum and kidney KIM-1, NAG, TNF-α, and IL-6, which may imply the potential anti-inflammatory effect of suppressing miR-34a using 3WJ-Kapt/anti-miR-34a.

At the molecular level, there are many interrelated and cross-talked mechanisms involved in the therapeutic effect of targeting miR-34a in CKD mice using 3WJ-RNPs containing anti-miR-34a. These mechanisms include the induction of the antifibrotic factors including α and β Klotho, SIRT1, and SMAD7, suppression of profibrotic factors including TGF-β, FGF2, WNT1, and β-catenin, and inhibition of renal inflammatory mediators (TNFα, and IL-6), as illustrated in the effect of 3WJ-Kapt/anti-miR-34a in CKD mice in the current study.

The CKD mice showed marked time-dependent up-regulation of the renal profibrotic pathways, including TGF-β, SMAD2, SMAD3, FGF2, and WNT/β-catenin pathways. The same mice showed suppressed renal expression of the antifibrotic pathways, including α and β Klotho, SMAD7, and SIRT1 at both mRNA and protein levels. In our study we chose to assyed the protein levels of the three important mediators of renal fibrotic pathway; TGF-β1, SMAD2, SMAD3, and klotho protein using specific mouse ELISA kits due to its high sensitivity, quantitative precision, and suitability for high-throughput analysis, which aligned with our study’s objectives. However, conduct additional confirmatory Western Blot experiments will be more informative.

The WNT/β-catenin pathway is one of the crucial signaling pathways that is significantly activated in CKD mice. During embryogenesis, the WNT/β-catenin pathway is important for nephron development. In adult kidneys, this pathway remains inactive. However, the pathway can be activated after renal injury [41, 42]. It was demonstrated that the accumulated free β-catenin is associated with destroying the epithelial integrity [43]. The prolonged stimulation of the WNT/β-catenin pathway contributes to kidney fibrosis by regulating the expression of downstream mediators that activate fibroblasts which are the primary driving force for renal fibrosis [44]. The WNT/β-catenin pathway enhances the pro-fibrotic effect of the TGF-β signaling pathway [45]. Increased FGF2 levels also confirm its involvement in causing interstitial fibrosis and glomerulosclerosis [46]. Effects that were reversed by 3WJ-Kapt/anti-miR-34a.

The correlation results of the present study indicated mutual positive correlations between the expression of TGF-β, FGF2, WNT, and β-catenin pointing to the interweaving of these profibrotic pathways to promote renal fibrosis. Normally, these pathways are controlled and counteracted by several interacting proteins including, Klotho, SIRT1, and SMAD7 that are markedly downregulated in CKD mice. SMAD7 has shown an intersection with the TGF-β profibrotic pathway, where it acts as a competitor of SMAD2 and SMAD3, the TGF-β downstream molecules, resulting in the inhibition of TGF-β signaling pathway [47], where enhanced renal expression of SMAD7 and reduced content of SMAD 2 and SMAD3 in the present study with 3WJ-Kapt/anti-miR-34a may partially explain the reduced TGF-β activity.

Both α and β Klotho are critical genes that control kidney homeostasis and aging and are considered ideal intervention targets for many renal diseases and even extrarenal complications [48]. They play important antifibrotic roles by inhibiting excessive inflammation and oxidative stress, and their deficiency promotes renal fibrosis [49]. Klotho is an essential negative regulator of canonical WNT/β-catenin signaling as Klotho’s extracellular domain suppresses WNT/β-catenin signaling by interacting with numerous WNT ligands [50] and inhibits renal fibrosis. α Klotho is extensively expressed in the kidney, specifically in the tubular epithelium of normal adult kidneys, and serves as a co-receptor for FGF2 [51]. Also, Klotho proteins simultaneously suppress other growth factor signaling pathways, including FGF2 and TGF-β [52]. The correlation results of the present study confirm such a negative association between α and β Klotho expression and the expression of the components of the profibrotic pathways: TGF-β, FGF2, WNT1, and β-catenin. Moreover, Klotho is an anti-inflammatory modulator that negatively downregulates the NF-κB pathway, resulting in reduced expression of the proinflammatory gene [38]. Hence, the restored expression and protein content of renal Klotho level in CKD mice by 3WJ-Kapt/anti-miR-34a in this study is considered one of the main pathways in reversing renal fibrosis and inflammation.

Another important antifibrotic agent against CKD is SIRT1 [19]which has been restored in the renal tissue of CKD mice upon treatment with anti-miR34a 3WJ nanoparticles. SIRT1 induces deacetylation and deactivation of SMAD3 and SMAD4, thereby inhibiting the profibrotic response of TGF- β1 in vitro and in vivo models of renal fibrosis [53–55]. SIRT1 inhibits NF-kB and TNF-α induced cytokine production in fibroblast cells and the expression of other proinflammatory genes [56]. SIRT1 also protected mouse renal medullary interstitial cells from oxidative stress [57]. The marked reduction of SIRT expression in CKD mice may be explained by the enhanced TGF-β pathway which induces miR-373 expression that targets SIRT1 and enhanced renal fibrosis [58]. The prominent role of declined SIRT1 expression in the induction of CKD in our study was confirmed by the correlation studies which indicated its negative association with KIM-1, NAG, TGF-β, FGF2, WNT1, β-catenin, TNF-α, and IL-6, and positive association with the antifibrotic markers, α and β klotho and SMAD. An effect that was reversed upon the 3WJ-Kapt/anti-miR-34a administration.

From previous studies and our results, we can consider miR-34a as one of the main regulators that master the pathogenesis of CKD and renal fibrosis because of its wide targets and pleiotropic effects. MiR-34a is involved in many cellular processes like growth, differentiation, and metabolism by negatively regulating typical target genes [59]. The induced expression of miR-34a in our CKD model could explain the marked suppression of Klotho and SIRT1 genes that result in enhanced expression of TGF-β, FGF2, WNT1, and β-catenin. This has been confirmed by its negative correlation with all antifibrotic factors and positive correlation with profibrotic factors, inflammatory markers, and CKD markers (KIM-1, NAG). As a central player in this pathway, miR-34a is a potential therapeutic target of CKD [59]. This encouraged us to select anti-miR-34a to be carried on the therapeutic domain of the constructed 3WJ-Kapt/anti-miR-34a in this study.

As previously reported, inhibitors of miR-34a showed amelioration of renal fibrosis [60], and improved liver fibrosis [61], while subcutaneous injection of LNA-antimiR-34a enhanced cardiac function and reduced myocardial fibrosis [62]. Targeting CKD with 3WJ-Kapt/anti-miR-34a in this study was associated with rapid downregulation of the expression level of miR-34a, reaching a completely normal level after the 1^st^ week of treatment followed by complete normalization of the renal expression of TGF-β, SMAD2/3, klotho, WNT1, and β-catenin. These data align with the previous studies, where therapeutic targeting of TGF-β and WNT/β-catenin pathway ameliorated fibrosis in rodent models of CKD [63], and reduced fibroblast gene activation, potentially improving fibrosis [64]. The prolonged therapeutic effect of RNPs 3WJ-Kapt/anti-miR-34a may be explained by using LNA and modified strands also it may be the miR34 knockdown affects downstream pathways and becomes functional along the experimental period. At the protein level, the 3WJ-Kapt/anti-miR-34a showed a marked reduction of renal SMAD2/3 and elevation of klotho proteins all of which completely normalized after 4 weeks of treatment while its effect on the TGF-β was mild and stable during the treatment period which may confer that its effects are mainly the downstream components of the TGF-β signaling pathway. These data are consistent with the molecular targets of miR-34a which mau imply that the observed effects may mediated mainly through the ant-miR-34a domain. Howevere, the possible biological effects of other domains of the used RNPs may exist; the aptamer, can not be excluded. As the DNA aptamer alone may interfere with the biology of the target cells, it will be better to consider further studies using Kapt-RNPs with no therapeutic domain to explore their possible biological effects.

The present study provides preliminary and pioneer evidence for the promising treatment of CKD and renal fibrosis through targeting miR-34a in the renal tissue using properly designed and constructed multifunctional 3WJ-RNPs comprising anti-miR-34a stabilized by 2′-F ribonucleotides and LNA and targeted to kidney using DNA aptamer. Our results confirmed that the suppression of kidney miR-34a resulted in the induction of antifibrotic pathways and suppression of profibrotic pathways. These molecular improvements were associated with marked amelioration at the histological level. More importantly, the prepared RNPs have shown very low or no toxicities in the main organs. From these findings, we conclude that the 3WJ-RNPs have the potential to be employed in clinical applications as a customized therapeutic delivery system to treat a variety of disorders in vivo due to the simplicity and flexibility of modification of each RNA module. Designing and constructing multifunctional renal-targeted 3WJ-RNPs containing anti-miR-34a is feasible and opens a new era of therapeutic approaches.

## Supplementary information

Supplementary file

## Data Availability

All data produced in this study can be available from the corresponding author upon a reasonable request.
