# Supplementary material for: Aptamer-targeted anti-miR RNA construct based on 3WJ as a new approach for the treatment of chronic kidney disease in an experimental model
Source: Gene Ther. 2025 Jun 14;32(4):359–75. doi: 10.1038/s41434-025-00544-7 (PMC12310521; doi:10.1038/s41434-025-00544-7)
Supplement: Supplementary file 1 — Supplementary file [file 41434_2025_544_MOESM1_ESM.docx]

**Aptamer-Targeted anti-miR RNA construct based on 3WJ as a new approach for the treatment of chronic kidney disease in an experimental model**

**Florescent scanning**

Fluorescence spectra of the RNPs were determined for dry and buffer-dispersed particle preparations, as well as in contact with fixed tissue sections from control mice using spectral imaging fluorescence microscopy. The extrinsic spectra emitted by the particles in contact with the corresponding tissue section from control mice were used as positive controls. As a negative control, the intrinsic autofluorescence spectra of the corresponding sections of the control organs were used.

**Kidney and liver function tests**

Serum ALT and AST activities were determined using reagents obtained from BioMed Diagnostics INC (USA), absorbance was measured at 340 nm. Urea and Creatinine were determined using reagents obtained from BioMed Diagnostics INC (USA), absorbance was measured at 570 nm and 510 nm, respectively.

Serum erythropoietin was assayed using a Mouse ELISA kit (Cloud Clone Gorp, USA, Cat. No. SEA028Mu); and Blood hemoglobin was measured using a Sysmex count full automation hematology analyzer **[1].** Malondialdehyde in the whole homogenate of the tissue samples was determined according to the method of Draper, H.H. & Hadley **[2].**

**Renal injury and inflammatory markers**

The excised kidneys were homogenized in RIPA buffer and the total protein was determined by a modification of Lowry methods **[3].**  The supernatants were used for the determination of KIM-1 and NAG in serum and kidney tissues using a mouse KIM-1 ELISA (eBioscience, USA, Cat. No. BEK1131), mouse NAG ELISA (eBioscience, USA, Cat. No. BZEK2142); respectively according to the instructions of the manufacturer. SMAD2 and SMAD3 were assayed in kidney tissues using a mouse-specific kit (LSBio, USA, Cat. No. LS-F19435, and LS-F6417, respectively) according to the instructions of the manufacturer. Klotho protein and Transforming growth Factor β (TGF-β) were assayed using mouse-specific kits (Cusabio, USA, Cat. No. CSB-E14362m, and CSB-E04726m, respectively). TNF-α and IL-6 were also assayed in kidney tissues using a mouse TNF-α ELISA (eBioscience, USA, Cat. No. BEK1213), mouse IL-6 ELISA (eBioscience, USA, Cat. No. BEK1109); respectively according to the instructions of the manufacturer.

**Gene expression analysis using quantitative real-time polymerase chain reaction**

Total RNA was extracted using miRNeasy Mini Kit (Qiagen, Germany, Cat. No. 217004). Messenger RNAs and miRNA were isolated from the kidney tissue according to the manufacturer's instructions. Quantitative analysis of miR-34a, TGF-β, Fibroblast Growth Factor 2 (FGF2), Suppressor of Mothers against Decapentaplegic (SMAD7), β-Klotho, α-Klotho, Wingless-related integration site (WNT1), β-catenin, and SIRT1 gene expression in kidney tissues was performed using qRT-PCR. First, the total RNA was isolated from the tissues, then the isolated RNA was reverse transcribed into complementary DNA (cDNA) using RevertAid First Strand cDNA Synthesis Kit (Thermo Scientific, Cat. No. #K1622) according to the manufacturer's instructions. Relative quantification of gene expression was performed using ViPrime PLUS Taq qPCR Green Master Mix (Viviantis Technologies, Malaysia, Cat. No. QlMM12).

Quantitative PCR amplification conditions were adjusted as an initial denaturation at 95°C for 5 min and then 45 cycles of PCR for amplification as follows: denaturation at 94 °C for 20 s, annealing at 55 °C for 20 s, and extension at 70 °C for 15 s. Data was collected using Bio-Rad CFX Mastero version 2.3 (Bio-Rad, Inc USA). The relative expression was quantified relative to the expression of the reference gene (18s rRNA) in the same sample and mir-34a to that of U6 using ΔΔCt method. The primers used are presented in **Table 1.** The relative change in mRNA expression in samples was estimated using the 2^-ΔΔCt^ method.

**Table (1): Mouse Primer sequences.**

| **Gene** | **Accession number** | **Primer sequence** | |
| --- | --- | --- | --- |
| **TGF-β1** | **NM_011577** | **F:** | 5'- TGATACGCCTGAGTGGCTGTCT -3' |
|  |  | **R:** | 5'- CACAAGAGCAGTGAGCGCTGAA -3' |
| **FGF2** | **NM_008006** | **F:** | 5’- AAGCGGCTCTACTGCAAGAACG -3' |
|  |  | **R:** | 5′- CCTTGATAGACACAACTCCTCTC -3' |
| **SMAD7** | [**NM_030858.2**](https://www.ncbi.nlm.nih.gov/entrez/viewer.fcgi?db=nucleotide&id=1937369551) | **F:** | 5'-GTCCAGATGCTGTACCTTCCTC -3' |
|  |  | **R:** | 5'- GCGAGTCTTCTCCTCCCAGTAT -3' |
| **β-Klotho** | **NM_031180** | **F:** | 5′- GAAAGAGTCCACGCCAGACATG - 3' |
|  |  | **R:** | 5'- CAGGTGAGGATCGGTAAACTGC -3' |
| **α-Klotho** | **NM_013823** | **F:** | 5'- CCTCCTTTACCTGAAAACCAGCC -3' |
|  |  | **R:** | 5'- CCACAGATAGACATTCGGGTCAG -3' |
| **WNT1** | **NM_021279** | **F:** | 5'- CGAGAGTGCAAATGGCAATTCCG -3' |
|  |  | **R:** | 5'- GATGAACGCTGTTTCTCGGCAG -3' |
| **SIRT1** | **NM_019812** | **F:** | 5'- GGAGCAGATTAGTAAGCGGCTTG -3' |
|  |  | **R:** | 5'- GTTACTGCCACAGGAACTAGAGG -3' |
| **β-catenin** | **NM_007614** | **F:** | 5'- GTTCGCCTTCATTATGGACTGCC -3' |
|  |  | **R:** | 5'- ATAGCACCCTGTTCCCGCAAAG -3' |
| **18S rRNA** | **NR_046237.2** | **F:** | 5'- GTAACCCGTTGAACCCCATT -3' |
|  |  | **R:** | 5'- CAAGCTTATGACCCGCACTT -3' |

**Table (3): Correlation coefficient between different parameters in CKD mice untreated, core three-way junction (3WJ) or therapeutic three-way junction (3WJ-Kapt/anti-miR-34a) treated.**

|  | **miR-34a** | **FGF2** | **α Klotho** | **β Klotho** | **SMAD7** | **WNT1** | **β Catenin** | **SIRT1** | **TGF-β** |
| --- | --- | --- | --- | --- | --- | --- | --- | --- | --- |
| **MDA** | 0.677^**^ | 0.607^**^ | -0.507^**^ | -0.348^**^ | -0.107 | 0.485^**^ | 0.511^**^ | -0.466^**^ | 0.455^**^ |
| **IL-6** | 0.620^**^ | 0.477^**^ | -.687^**^ | -0.507^**^ | -0.338^**^ | 0.556^**^ | 0.579^**^ | -0.564^**^ | 0.573^**^ |
| **TNF-α** | 0.731^**^ | 0.584^**^ | -.684^**^ | -0.583^**^ | -0.287^*^ | 0.572^**^ | 0.626^**^ | -0.630^**^ | 0.630^**^ |
| **Serum**  **KIM1** | 0.604^**^ | 0.411^**^ | -.521^**^ | -0.508^**^ | -0.537^**^ | 0.637^**^ | 0.580^**^ | -0.532^**^ | 0.654^**^ |
| **Renal**  **KIM1** | 0.649^**^ | 0.725^**^ | -.648^**^ | -0.537^**^ | -0.493^**^ | 0.662^**^ | 0.792^**^ | -0.540^**^ | 0.741^**^ |
| **Serum**  **NAG** | 0.737^**^ | 0.491^**^ | -.620^**^ | -0.464^**^ | -0.397^**^ | 0.549^**^ | 0.585^**^ | -0.499^**^ | 0.593^**^ |
| **Renal**  **NAG** | 0.703^**^ | 0.694^**^ | -.650^**^ | -0.501^**^ | -0.402^**^ | 0.645^**^ | 0.739^**^ | -0.535^**^ | 0.640^**^ |
| **miR-34a** | 1 | 0.525^**^ | -.604^**^ | -0.531^**^ | -0.271^*^ | 0.720^**^ | 0.676^**^ | -0.677^**^ | 0.635^**^ |
| **FGF2** | 0.525^**^ | 1 | -.466^**^ | -0.233 | -0.015 | 0.482^**^ | 0.736^**^ | -0.267^*^ | 0.394^**^ |
| **α Klotho** | -0.604^**^ | -0.466^**^ | 1 | 0.654^**^ | 0.313^*^ | -0.533^**^ | -0.520^**^ | 0.588^**^ | -0.565^**^ |
| **β Klotho** | -0.531^**^ | -0.233 | .654^**^ | 1 | 0.492^**^ | -0.433^**^ | -0.346^**^ | 0.689^**^ | -0.566^**^ |
| **SMAD7** | -0.271^*^ | -0.015 | .313^*^ | 0.492^**^ | 1 | -0.286^*^ | -0.332^**^ | 0.348^**^ | -0.584^**^ |
| **WNT1** | 0.720^**^ | 0.482^**^ | -.533^**^ | -0.433^**^ | -0.286^*^ | 1 | 0.642^**^ | -0.440^**^ | 0.553^**^ |
| **β Catenin** | 0.676^**^ | 0.736^**^ | -.520^**^ | -0.346^**^ | -0.332^**^ | 0.642^**^ | 1 | -0.398^**^ | 0.590^**^ |
| **SIRT1** | -0.677^**^ | -0.267^*^ | .588^**^ | 0.689^**^ | 0.348^**^ | -0.440^**^ | -0.398^**^ | 1 | -0.572^**^ |
| **TGF-β** | 0.635^**^ | 0.394^**^ | -.565^**^ | -0.566^**^ | -0.584^**^ | 0.553^**^ | 0.590^**^ | -0.572^**^ | 1 |
| **SMAD2** | 0.780^**^ | 0.550^*^ | -0.60^*^ | -0.52^*^ | -0.390 | 0.654^*^ | 0.565^**^ | -0.654^**^ | 0.632^*^ |
| **SMAD3** | 0.777^**^ | 0.560^**^ | -0.564^*^ | 0.490^**^ | -0.290 | 0.567^**^ | 0.567^*^ | -0.654^**^ | -0.567^*^ |

*correlation study was checked by Pearson correlation*

****:*** *significant correlation p<0.05.*

*****:*** *significant correlation p<0.01.*

**
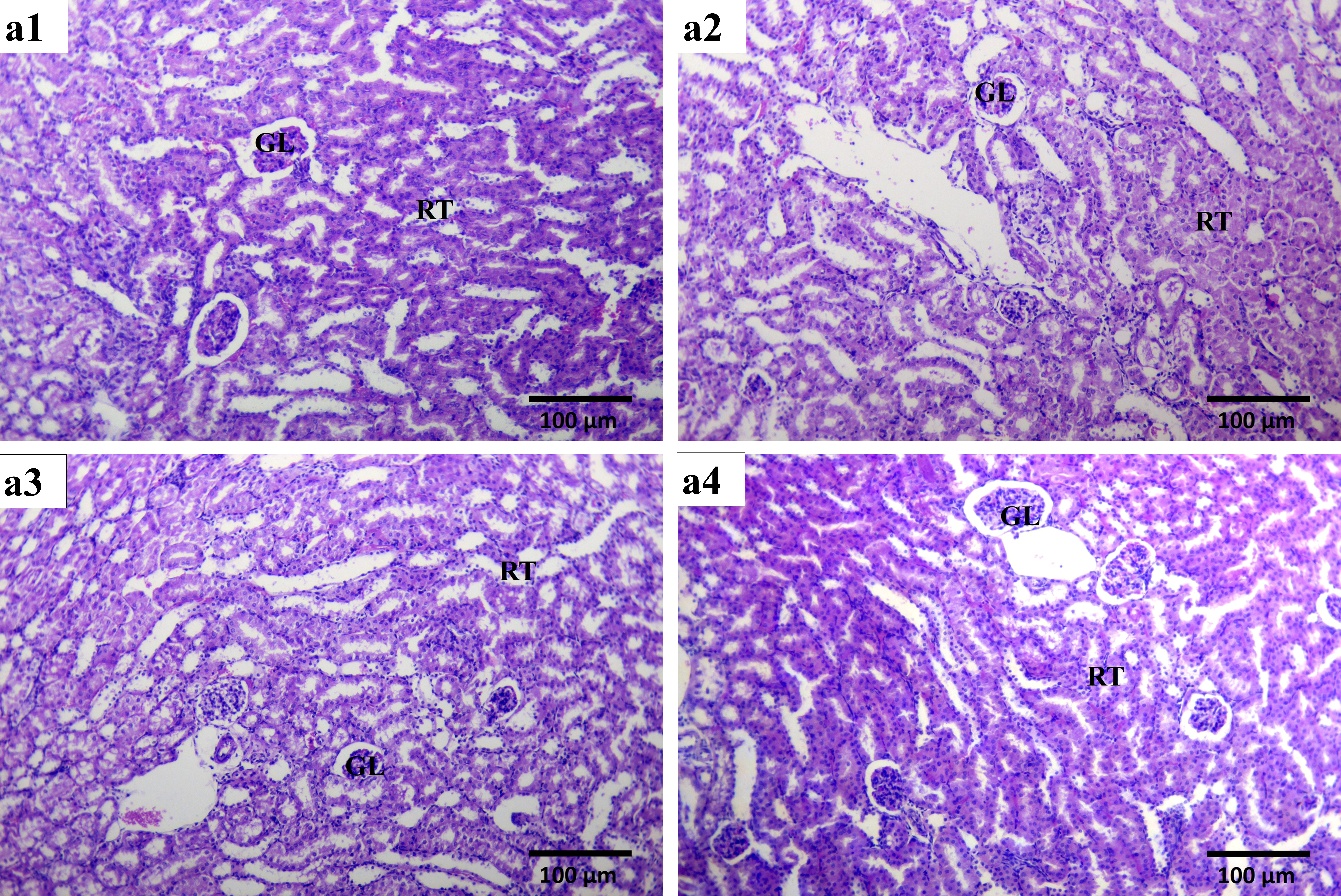
**

**Fig. 6 (a1-a4):** **Representative photomicrographs of mice kidneys** from the control group **at different experimental periods** (H&E stain, x100). a1:one week, a2:two weeks, a3: three weeks and a4: four weeks treatments. [Normal renal glomeruli **(GL)** and renal tubule **(RT)**].


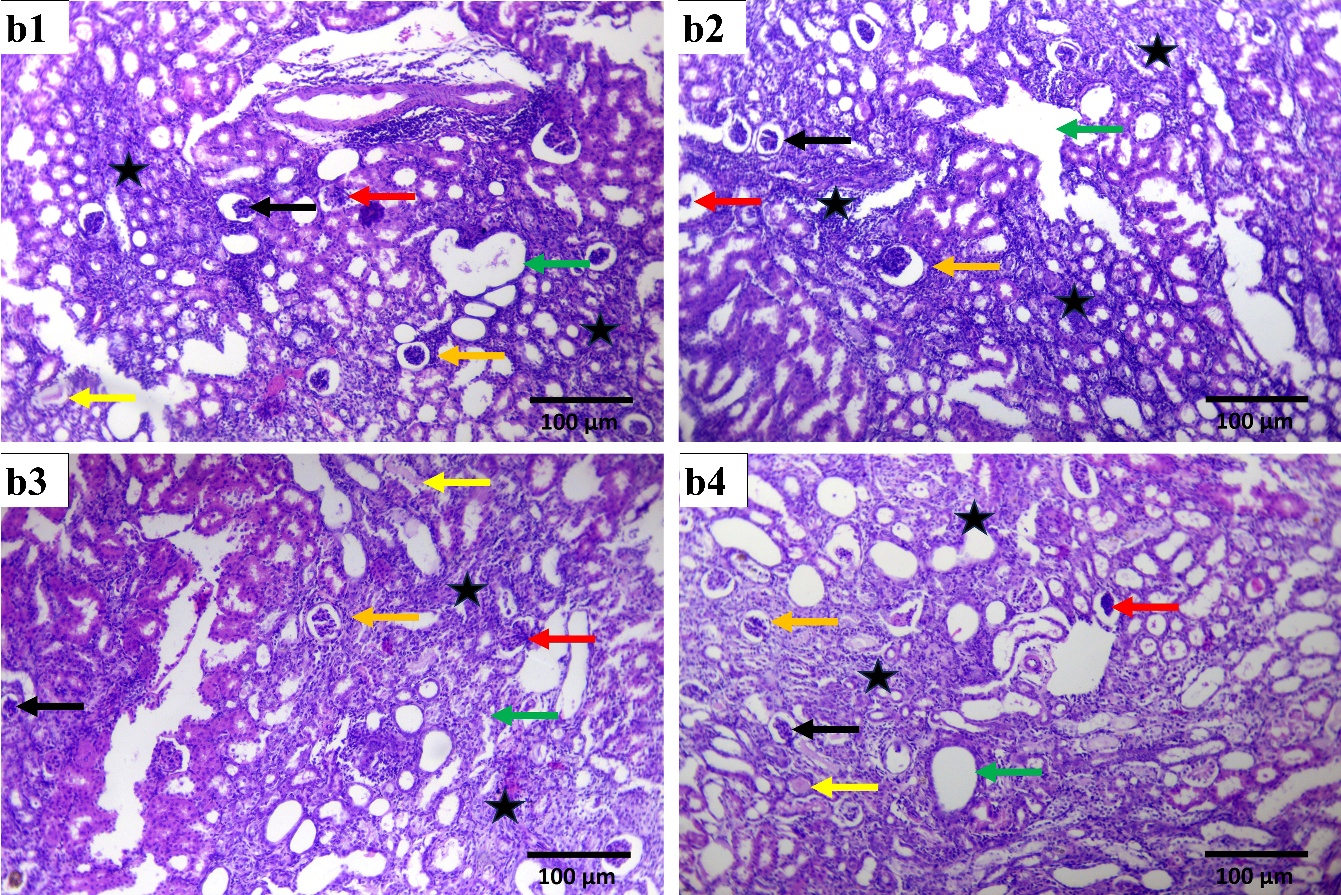


**Fig. 6 (b1-b4):** **Representative photomicrographs of** mice **kidneys** from the CKD group **at different experimental periods** (H&E stain, x100). b1:one week, b2:two weeks, b3: three weeks and b4: four weeks treatments. [dilatation of the renal tubular lumen **(green arrow)**, fibrous tissue proliferation and interstitial mononuclear inflammatory cell infiltrations **(star),** atrophied glomeruli **(black arrow),** necrotic glomeruli **(red arrow),** periglomerular fibrosis **(orange arrow)** and intratubular cast formation **(yellow arrow)**]**.**


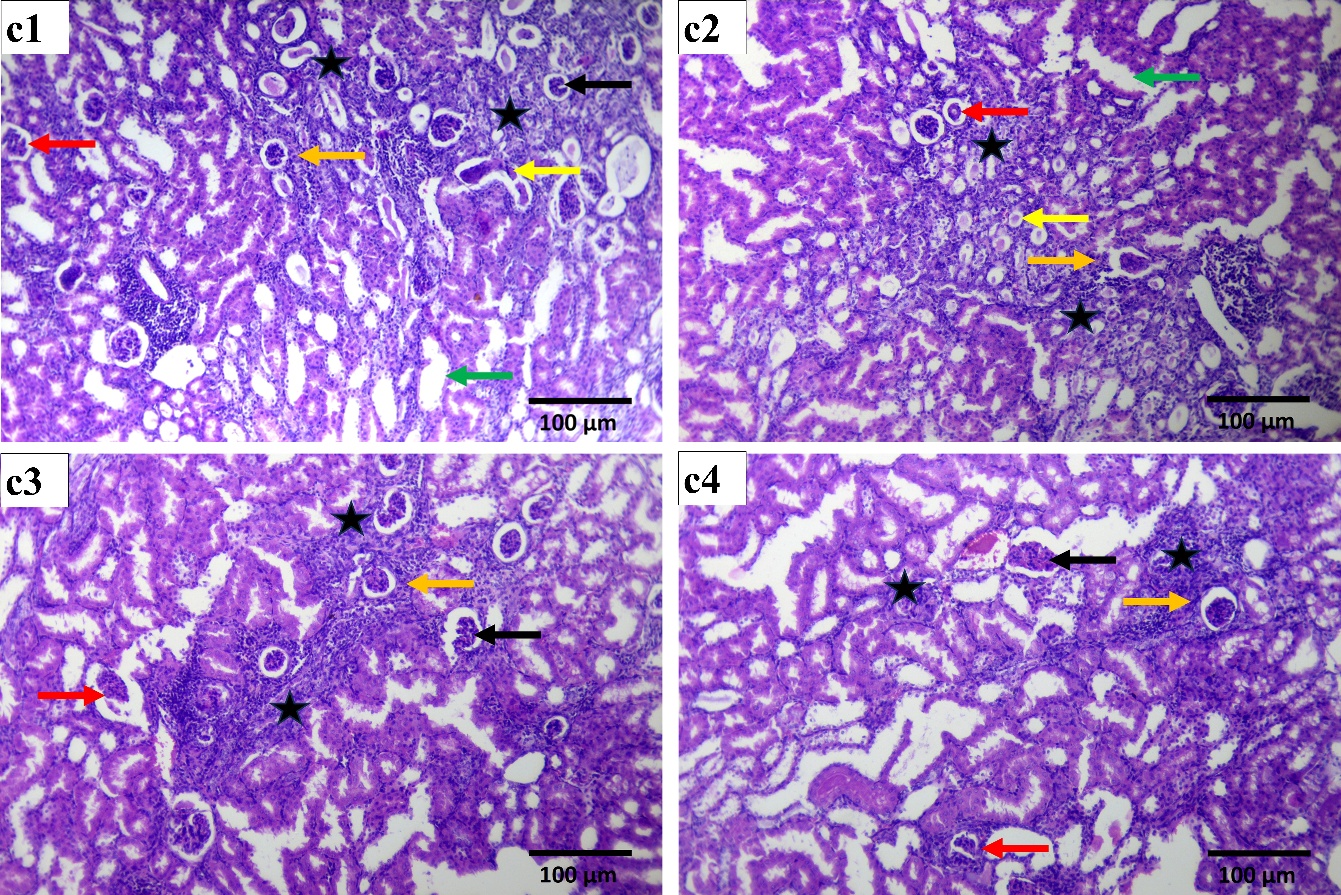


**Fig. 6 (c1-c4):** **Representative photomicrographs of** mice **kidneys** from the 3WJ group **at different experimental periods** (H&E stain, x100). c1:one week, c2:two weeks, c3: three weeks and c4: four weeks treatments. [dilatation of the renal tubular lumen **(green arrow)**, fibrous tissue proliferation and interstitial mononuclear inflammatory cell infiltrations **(star),** atrophied glomeruli **(black arrow),** necrotic glomeruli **(red arrow),** periglomerular fibrosis **(orange arrow)** and intratubular cast formation **(yellow arrow)**]**.**


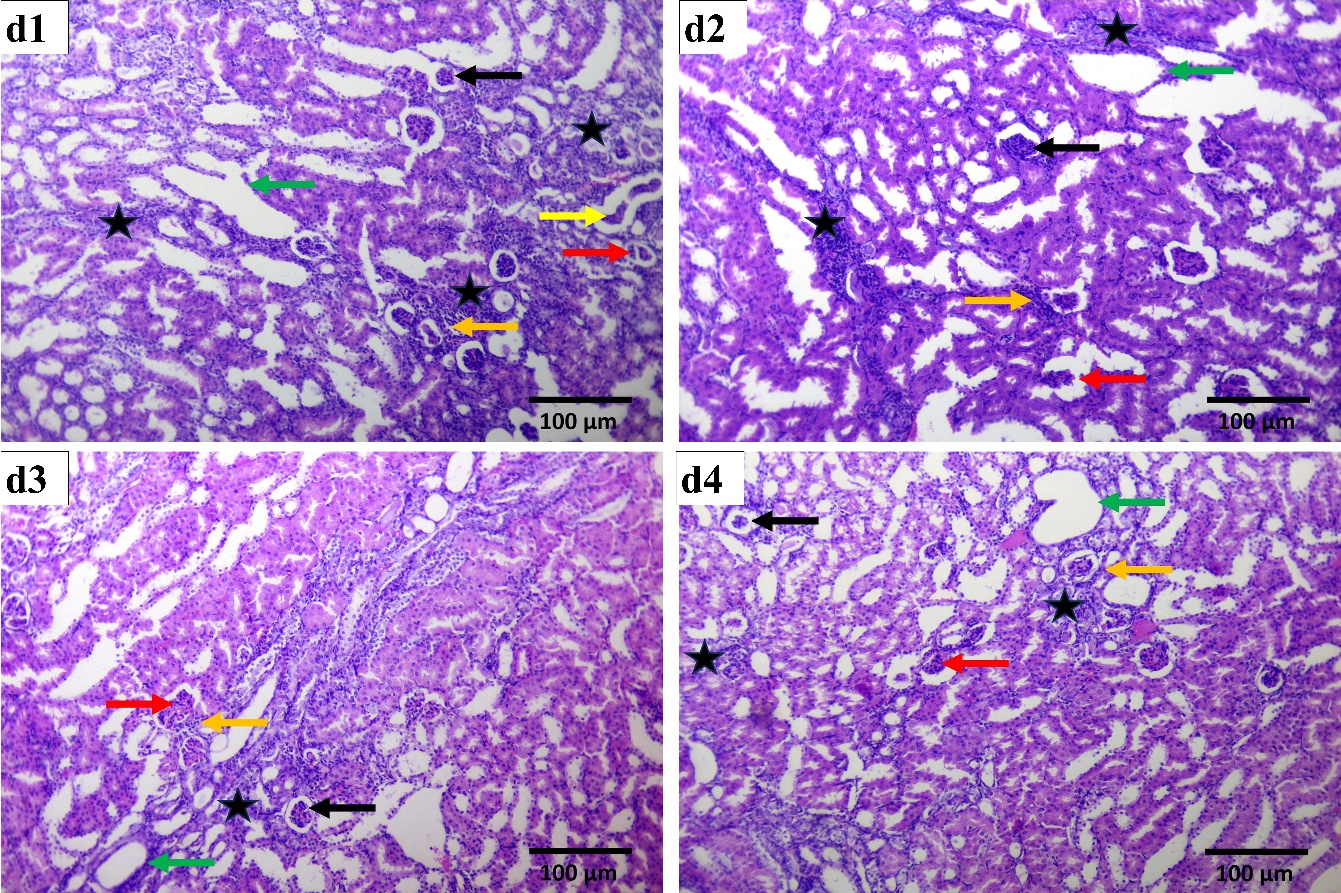


**Fig. 6 (c1-c2):** **Representative photomicrographs of** mice **kidneys** from the 3WJ-Kapt/anti-miR-34a group **at different experimental periods** (H&E stain, x100). d1:one week, d2:two weeks, d3: three weeks and d4:four weeks treatments. [dilatation of the renal tubular lumen **(green arrow)**, fibrous tissue proliferation and interstitial mononuclear inflammatory cell infiltrations **(star),** atrophied glomeruli **(black arrow),** necrotic glomeruli **(red arrow),** periglomerular fibrosis **(orange arrow)** and intratubular cast formation **(yellow arrow)**]**.**

References

1. Grillone R, Grimaldi E, Scopacasa F, Dente B. Evaluation of the fully automated hematological analyzer M indray BC 6800: comparison with H oriba ABX P entra DX 120. International Journal of Laboratory Hematology. 2014;36(4):e55-e8.

2. Draper HH, Hadley M. Malondialdehyde determination as index of lipid Peroxidation. In: Draper HH, Hadley M, editors. Methods in enzymology. London: Elsevier; 1990. p. 421-31.

3. Lowry O, Rosebrough N, Farr AL, Randall R. Protein measurement with the Folin phenol reagent. Journal of biological chemistry. 1951;193(1):265-75.

4. Bancroft JD, Layton C, Suvarna SK. Bancroft's theory and practice of histological techniques. 3^rd^ ed. London: Churchill Livingstone Elsevier; 2013.

5. Zhang X-M, Min X-R, Li D, Li B, Xie H-X, Liu R, et al. The protective effect and mechanism of piperazine ferulate in rats with 5/6 nephrectomy-caused chronic kidney disease. Naunyn-Schmiedeberg's Archives of Pharmacology. 2024:1-15.
